# A naturalized gut microbiome interacts with dietary fibers to protect against colonic inflammation

**DOI:** 10.1080/19490976.2026.2649435

**Published:** 2026-03-28

**Authors:** Signe Birkeland, Ingerid Rohde Mæhlum, Marte Senneset, Ingrid Wik Taxerås, Lars Snipen, Henriette Markov Arnesen, Preben Boysen, Harald Carlsen

**Affiliations:** a Faculty of Chemistry, Biotechnology and Food Science, Norwegian University of Life Sciences (NMBU), Ås, Norway; b Faculty of Veterinary Medicine, Norwegian University of Life Sciences (NMBU), Ås, Norway; c Department of Research, Østfold Hospital Trust, Sarpsborg, Norway

**Keywords:** Feralized mice, naturalized mice, gut microbiome, DSS-induced colitis, dietary fiber, inflammatory bowel disease

## Abstract

“Feralized” mice, housed in farmyard-type environments, show a matured immunophenotype, altered intestinal barrier, and a shifted gut microbiome compared to conventionally housed laboratory mice. Since dietary fibers support gut health in part by microbial fermentation into immunomodulatory short-chain fatty acids, we hypothesized that feralization influences the intestinal barrier by enhancing the fiber-degrading properties of the microbiome. We explored whether susceptibility to low-grade dextran sulfate sodium-induced colitis differed between feralized and clean laboratory mice fed diets high or low in fermentable fibers. Feralized mice were protected against colitis, displaying low disease scores and biomarkers of inflammation in feces, plasma, and liver; and altered colonic mucosal gene expression, compared to clean mice. This protection was strongest with a fiber-rich diet, which, in contrast, worsened colitis in clean mice. Transfer of fecal microbiota from feralized mice to clean recipients conferred colitis protection. Fecal metagenome-assembled genomes revealed that the fiber-rich diet enriched the microbiome with predicted genes encoding fiber-degrading enzymes, while the low-fiber diet promoted mucin-degrading enzyme genes. However, the dominant microbial species contributing to these functions differed between feralized and laboratory mice. Differential abundance of bacterial taxa in feralized and laboratory mice further identified potential microbial modulators of colitis that merit targeted investigation in future studies. Overall, these findings suggest that fibers affect intestinal inflammation in a microbiota-dependent manner, underscoring the complex interplay between diet and microbiota in disease development.

## Introduction

The prevalence of inflammatory bowel disease (IBD), including Crohn's disease (CD) and ulcerative colitis (UC), continues to increase in westernized regions such as Northern Europe, North America, and Australia.^1^ While the mechanisms behind IBD development remain poorly understood, risk factors include genetic alterations (e.g., mutations in nucleotide oligomerization domain 2 (*NOD2*) and autophagy-related 16-like 1 (*ATG16L1*)), gut microbiota composition, environmental exposures, and lifestyle factors such as diet.^1‐4^ Cleaner living and urbanization are associated with increased incidence rates of IBD as well as other immune-related diseases,^5^ and the hygiene hypothesis and its relatives, including the “old friends hypothesis”, have been proposed to partly explain this rise.^6^ “Old friends” describes the (micro)organisms with which we have co-evolved that may be essential for optimal development and function of our immune system. Laboratory mice, compared to wild house mice (*Mus musculus musculus*), lack some of these “old friends”,^7^ which might explain the persistent limitations to confident translation of results from preclinical mouse trials to humans.^8^^,^^9^ In view of this hypothesis, the concept of “naturalizing” laboratory mice emerged, in which the aim is to provide a more naturalistic setting for mouse experiments.^10‐13^ We have previously established the “feralized” mouse model, where laboratory mice are provided a farmyard-like habitat.^10,14‐16^ Across feralized mice and other naturalization methods (rewilding, co-housing, fetal transfer to wild mice, and infection models),^17^^,^^18^ mice display altered immunophenotypes,^11^^,^^12^^,^^19^^,^^20^ changes in the gut microbiota,^12^^,^^16^ protection against diseases including colorectal cancer (CRC),^14^ bacterial infections,^21^^,^^22^ diet induced obesity,^23^ and more appropriate responses against vaccines and diseases compared to conventionally housed laboratory mice.^7^^,^^12^^,^^24^ Regardless of the approach, naturalized mice provide valuable insights into how microbial exposure from the living environment influences both mucosal and systemic immune homeostasis.

Diet is a major lifestyle factor that affects both the gut microbiota and the risk of developing diseases associated with the gut. In particular, the intake of diets high in dietary fibers (DFs), is linked to maintenance of the intestinal barrier and a reduced risk of IBD, cardiovascular disease, CRC, and diabetes.^25‐29^ The protective effects of DFs, which are complex carbohydrates not digested in the human small intestine,^30^ are largely attributed to their role as a substrate of microbial fermentation in the colon. This can alter the composition of the gut microbiota and produce short-chain fatty acids (SCFAs) that have numerous biological functions, including lowering the pH and modulating the intestinal immune compartment.^31‐34^ Microbial species in the gut carry a diverse array of genes encoding carbohydrate-active enzymes (CAZymes) that determine how effectively DFs are utilized, both through direct digestion and by supporting other microbes via cross-feeding.^35^ Microbes with a greater capacity to metabolize complex carbohydrates are also more likely to successfully colonize the gut.^36^ As a result, the microbiome composition plays a crucial role in how the host responds to DF intake, which simultaneously affects the microbiome composition.

The mechanisms by which a naturalized microbiota modulates the intestinal barrier and gut immunity remain elusive. In this study, we hypothesized that naturalizing laboratory mice could alter the DF-degrading properties of the colonic microbiome, potentially uncovering how environmentally derived microbes influence gut barrier properties. To explore this, we compared disease outcomes following low-grade colitis induced with 1% dextran sulfate sodium (DSS) in feralized and clean laboratory mice that were fed diets enriched or deprived of fermentable DFs.

## Materials and methods

### Animals and experimental setup

**Figure 1.**
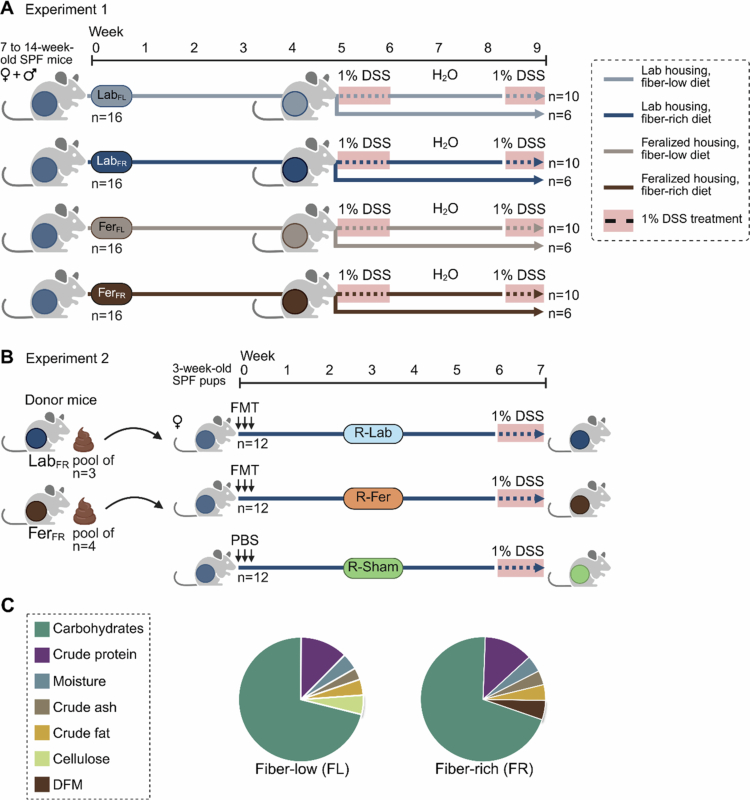
Experimental design for (A) experiment 1 and (B) experiment 2. DSS treatment period highlighted in red. (C) Composition of mouse diets: left, fiber-low (FL) and right, fiber-rich (FR). DFM consisted of rye bran (50%), wheat bran (35%), and oat bran (15%). Complete special diets compositions are listed in **Supplementary Table S1**. Lab, laboratory; Fer, feralized; FL, fiber low; FR, fiber rich; FMT, fecal microbiota transfer; DSS, dextran sodium sulfate; D, donor; R, recipient; SPF, specific pathogen free; DFM, dietary fiber mix.

In the first experiment, we obtained 64 locally bred transgenic B6-NF-κB^Luc^ and wild-type (WT) C57BL/6JRj mice (B6; purchased from Janvier Labs, Le Genest-Saint-Isle, France). B6-NF-κB^Luc^ mice express luciferase upon NF-κB (nuclear factor kappa-light-chain-enhancer of activated B cells) activation, made detectable by the injection of luciferin.^37^ The mice were bred in two cycles, resulting in 32 male and 32 female offspring aged 7 to 14 weeks, of which 43 were B6-NF-κB^Luc^ (16 males, 27 females) and 21 were WT (16 males, 5 females). The mice were randomly assigned to one of the four age- and sex-matched groups: Lab_FR_, Lab_FL_, Fer_FR_, or Fer_FL_ (*n* = 16 per group; Figure 1A). Mice were housed either in a microbially enriched environment by feralization (Fer) or a clean specific pathogen free (SPF) environment (Lab), and within each housing condition, fed either a fiber-low (FL) diet or a fiber-rich (FR) diet. Feralization was performed similarly as previously described,^10,14‐16^ enriching cages with organic plant soil (EAN: 7058782515260, Plantasjen, Norway; containing decomposed peat, sand, lime, clay, and hygienized chicken manure) and farm animal droppings from cow, sheep, pig, and poultry from an organic farm in south-eastern Norway (Ramme Gård, Vestby, Norway), and horse droppings from the Livestock Production Research Center (NMBU, Ås, Norway). Fresh feralizing material was added to the cages every second week to ensure continuous exposure to naturalistic environmental microbes. Mice were fed a standard rodent chow diet (Ssniff Spezialdiäten, #V1534-000) before introducing experimental diets. Experimental diets were either FR or FL (Figure 1C), with the AIN-93M diet serving as the FL diet,^38^ which contains low-fermentable cellulose (50 g/kg) as the source of DF.^31^ In the FR diet, cellulose was replaced by DFs originating from a mix of rye bran (50%), wheat bran (35%), and oat bran (15%), reflecting a typical Scandinavian intake of DF food sources.^39^ The amount of other dietary components in the fiber mix were adjusted accordingly to keep all other nutrients similar. For more details and diet intake, see **Supplementary Table S1** and **Figure S1**.

In the second experiment, fecal microbiota transfer (FMT) was conducted to explore the role of the microbiota. Fifteen female B6 mice (Janvier Labs) were housed and fed in a similar manner as for experiment 1 and used as donors of fecal material for FMT. Recipient pups for the FMT were obtained from a breeding of 16 female and eight male six-week-old B6 mice (Janvier Labs) staggered by one week, resulting in two batches of pups. Thirty-six female pups (recipients, R) from the breed were included. The dams and recipients were given the same FR diet used in experiment 1 and maintained under SPF conditions, and the dams were switched to the FR diet one week after the birth of pups. At weaning (age three weeks), pups were given FMT using fecal material from either Fer or Lab donor mice, or sham (*n* = 12 per group; R-Fer, R-Lab, R-Sham; Sham transfers consisted of anaerobic PBS + 10% glycerol) (Figure 1B). FMT was performed by giving single daily transfers by oral gavage using a 24 G olive-tip steel needle (AgnTho's) in the morning for three consecutive days (100 μL/dose; ~7.2 mg feces).

After five weeks of feralization and diet intervention in experiment 1, 10 mice in each group were administered 1% dextran sulfate sodium (DSS; MP Biomedicals, #160110) in the drinking water (w/w in autoclaved tap water) in two cycles of seven and five days, separated by a 16-d recovery period, to induce low-grade chronic colitis.^40^^,^^41^ Fresh bottles were provided every second day. The remaining six mice in each group served as controls and were given normal tap water. Five weeks after the FMT in experiment 2, recipients were challenged with one cycle of 1% DSS in the drinking water for seven days and subsequently euthanized. From what we observed in experiment 1, one cycle of DSS treatment was judged sufficient. In both experiments, mice were scored for Disease Activity Index (DAI) daily using standard scoring protocols that differed slightly between experiments, yet they evaluated similar parameters (**Supplementary Table S2**). In experiment 2, mice were given the analgesic tramadol (STADAPHARM, #4150049989753) in the drinking water (0.1 mg/mL) starting on day four of DSS treatment. Tramadol has been shown not to interfere with inflammation in the DSS model,^42^ and a low dose was chosen to maintain normal levels of water intake.^43^ Tramadol's interaction with the gut microbiome has, to our knowledge, not been examined.

All the mice were kept in individually ventilated cages (IVCs; Innovive Inc.), with 2–4 mice/cage, 25–27 °C ambient temperature, 12-hour light/dark cycle, and 40–60% relative humidity. Cages were enriched with sterile aspen woodchip bedding (Scanbur), mouse igloos, and running wheels. Food and water were provided *ad libitum*. Gloves were changed, and surfaces and equipment were disinfected with 70% ethanol between handling of mice in different groups.

The use of animals in these experiments was approved by the Norwegian Food Safety Authority (FOTS ID 29856 and 30292). Handling and care of mice was conducted in accordance with guidelines, terms and conditions determined by the EU Commission (directive 2010/63) and the Norwegian regulation concerning the use of animals for scientific purposes.

### Collection of fecal samples and preparation of fecal microbiota transfer inoculum

Fecal samples were collected on multiple occasions throughout both experiments. Mice were allowed to defecate in individual, sterile boxes in which fecal pellets were immediately collected using sterile forceps and placed in 2 mL screw cap tubes (SARSTEDT, #72.694.106), snap frozen in liquid nitrogen, and stored at −80 °C.

Fecal pellets collected from FMT donor mice were immediately placed in fresh, cold, reduced PBS (Dulbecco's phosphate-buffered saline (Biowest, #L0615) supplemented with 0.1% L-Cysteine hydrochloride as a reducing agent (Sigma-Aldrich, #C1276)) prepared as 1:20 w/v. The collection tube was only opened inside a box with dry ice to limit oxygen exposure and otherwise kept cold. The suspension was homogenized in Mixer Mill (MM 400, RETSCH; RT, 5 min, 30 Hz), transferred to an anaerobic chamber (Whitley A85 Workstation; 85% N_2_, 5% CO_2_, 10% H_2_) and filtered through a 70 μm cell strainer to remove crude particles. The filtrate was centrifuged for collection of bacteria (10 min, RT, 4000 × *g*), supernatant was discarded, and the pellet was resuspended in anaerobic PBS with 10% glycerol. Aliquots were snap frozen in liquid nitrogen and stored at −80 °C. Aliquots were thawed at 37 °C and briefly mixed by pipetting immediately prior to FMT.

### Termination of animal experiments and measurements of NF-κB activity

Mice were anesthetized with IP injection of ZRF cocktail: Zoletil Forte (Virbac; 3.2 mg/mL Zolazepam), Rompun (Bayer; 3.2 mg/mL Tiletamine, 0.45 mg/mL Xylazine), and Fentadon (Eurovet Animal Health; 2.6 μg/mL Fentanyl) in sterile, isotonic 0.9% NaCl; 10 μL/g body weight. Once under terminal anesthesia, mice were injected IP with 150 mg/kg luciferin (B6-NF-κB^Luc^; 10 μL/g body weight; Biosynth, #FL08608) or vehicle (WT B6; sterile DPBS, 10 μL/g body weight, Biowest, #0615), and 10 min after injection, blood was collected by cardiac puncture followed by cervical dislocation and sampling of tissues. The intestines and liver were subjected to *ex vivo* imaging by placing organs on a sterile, dark plastic sheet and placed in an IVIS Lumina II system (Revvity) for five min. Mucosa scrapings from the proximal half of the colons and liver tissues were collected for gene expression analysis, and ceca were cut into two pieces, snap frozen in liquid nitrogen and stored at −80 °C for SCFA and microbial analysis. In experiment 2, animals were euthanized similarly, excluding luciferin injection.

### Blood sampling and preparation of plasma

In experiment 1, blood samples were collected from the saphenous vein using Microvette® capillary blood collection tube with K_2_-EDTA (Sarstedt, #16.444.100) at the end of DSS-treatment cycle 1 (day seven). At the termination of both experiments, 500–1000 μL of blood was collected from the heart using a 25 G needle with 50 μL of 50  mM Na_2_-EDTA to prevent coagulation. In both experiments, plasma was obtained by centrifugation (10 min, 4 °C, 6000 × *g*) and collected, aliquoted, snap frozen in liquid nitrogen, and stored at −80 °C.

### Isolation of total RNA from colon mucosa and liver

Colon mucosa and liver tissue samples were handled similarly unless stated otherwise. For harvesting of colonic mucosa, the proximal to mid colon was flushed with cold PBS, cut longitudinally, and placed lumen-side up on a cold plate. The mucosa was scraped off using microscope slides treated with RNAse inhibitor (RNase*Zap*™, Invitrogen™, #AM9780) and immediately placed in RNA*later™* (Invitrogen™, #AM7021). Approximately 100 mg of the right liver lobe was cut off and placed in RNA*later*™ immediately after dissection. Tissues were kept at 4 °C for 24 h, then stored at −80 °C. On the day of RNA isolation, tissue samples were thawed on ice, and RNA was isolated from both tissues using NucleoSpin RNA Mini kit (Macherey-Nagel, #740955.250) following the manufacturer's protocol. Thirty milligrams of liver tissue was roughly minced using RNase inhibitor-treated scissors prior to homogenization in lysis buffer by passing the tissue through a needle (mucosa: 23 G, liver: 18 G). Purity and concentration of the eluted RNA were assessed using NanoDrop™ 2000 (Thermo Scientific™, #ND2000). The samples with A260/280 and A260/230 values in the ranges of 1.7–2.4 and 1.8–2.4, respectively, were accepted as pure RNA (**Supplementary Table S3**).

As DSS may inhibit PCR, RNA was purified by lithium chloride reprecipitation,^44^^,^^45^ and purity and concentration of the RNA extracts were examined anew by NanoDrop™ 2000. RNA integrity was assessed with RNA 6000 Nano kit (Agilent Technologies, #5067-1511) in Agilent 2100 Bioanalyzer with the 2100 Expert Software, providing an RNA integrity number (RIN value) ranging from 1 (degraded RNA) to 10 (intact RNA). RIN > 5 was considered sufficient RNA quality for subsequent qPCR analysis. All samples were considered of sufficient quality following purification.

### Relative gene expression analysis of liver tissue

Two-step quantitative reverse transcription PCR (RT‒qPCR) was applied to RNA isolated from the liver. Briefly, cDNA was prepared by reverse transcription using iScript cDNA Synthesis Kit (Bio-Rad, #1708891) with a final RNA concentration of 50 ng/μL in the reaction mix. The products were diluted 10X, resulting in a cDNA concentration of 5 ng/μL, combined with HOT FIREPol® EvaGreen® qPCR Supermix (Solis Biodyne, #08-36-00001) to 1 ng/μL, and qPCR and melting curve analysis run with duplicates in C1000 Touch™ Thermal Cycler with CFX96 Optical Reaction Module for Real-Time PCR Systems (Bio-Rad, #1841100, #1845096). Inter-run calibrators (IRC) were included using a pool of cDNA from a random selection of samples to account for plate variability. Quantification cycles (Cq) were determined with CFX Maestro™ Software (Bio-Rad, #12004110). Primer sequences and annealing temperatures are listed in **Supplementary Table S4**.

### Relative gene expression analysis of colonic mucosa

RT‒qPCR was applied to colonic mucosa RNA using Biomark™ HD (Standard Biotools Inc.) and an intestinal barrier gene panel of 37 target genes previously validated ^16^ following the manufacturer's protocol. Briefly, cDNA was prepared using Reverse Transcription Master Mix (Standard Biotools Inc., #100-6298) with 20 ng/μL RNA. Preamplification of cDNA was performed with 12 cycles using Preamp Master Mix (Standard Biotools Inc., #100-5580) with 5 ng/μL cDNA. Preamplified samples were cleaned with Exonuclease I (4 U/μL, Thermo Scientific, # EN0582) and diluted 10-fold. Analysis was performed using 96.96 Dynamic Array™ Integrated Fluidic Circuit (IFC, Standard Biotools Inc., #BMK-M-96.96) with a final template concentration of ~800 ng/μL. The primer sequences are listed in **Supplementary Table S4**.

### Measurements of fecal LCN2 and plasma levels of SAA and LBP by ELISA

Fecal pellets were homogenized in PBS with 0.1% Tween®20 (100 mg feces/mL; Sigma-Aldrich, #P1379) for 15 min at 30 Hz in Mixer Mill (Roche, experiment 1) or TissueLyser II (Qiagen; experiment 2), followed by centrifugation (10  min, RT, 12,000 rpm). Supernatants were diluted in PBS with 0.1% Tween®20 according to weight loss at termination (<1%, 1:200; 1–2%, 1:1000; 2–4%, 1:1500; >4%, 1:2000). Fecal LCN2 levels were measured with Mouse Lipocalin-2/NGAL DuoSet ELISA (R&D Systems, #DY1857-05). Plasma SAA was measured with Mouse Serum Amyloid A DuoSet ELISA (R&D Systems, #DY2948-05), and lipopolysaccharide binding protein (LBP) was measured with a Mouse LBP ELISA Kit (Biorbyt, #orb391103) following the manufacturers' protocols. Plasma samples destined for measuring SAA were diluted 1:1000 or 1:5000 using reagent diluent, whereas plasma samples destined for measuring LBP were diluted 1:10, 1:50, or 1:100 using sample diluent. Absorbance was measured at 450 nm with 540 nm for wavelength correction using SpectraMax M2® plate reader (Molecular Devices). All samples are presented as the sample-wise mean of technical duplicates. In cases where the signal was out of range on the low end (i.e., very little to no analyte), values were imputed randomly in R as a value between zero and the lowest valid value measured on the given plate.

### Short-chain fatty acid analysis

Cecal SCFA levels were analyzed by LabTek (Faculty of Biosciences, NMBU) using a TRACE 1300 series gas chromatograph (GC; Thermo Scientific) equipped with an autosampler, a flame ionization detector (FID), a split injector, and a Stabilwax DA column (30 m, 0.25 mm ID, 0.25 μm; Restek, #11023). Ceca were thawed on ice, and contents were extracted from the tissues and transferred to 200 μL of cold internal standard solution (2-methyl valeric acid in 5% formic acid). The samples were sonicated in cold water for 5  min and centrifuged (15  min, 15,000 × *g*, 4 °C). Supernatants were transferred to a spin column (45 kDa) for purification and centrifuged (15 min, 15,000 × *g*, 4 °C). The final supernatants were transferred to CG vials and analyzed in the GC-FID instrument. Finally, results were obtained using Chromeleon software (Thermo Scientific).

## 16S rRNA gene amplicon and shotgun metagenomic library preparation and sequencing

One fecal pellet and approximately 30 mg of cecal content were thawed on ice and mechanically lysed with acid-washed glass beads (0.2 g < 106 μm + 0.2 g 425–600 μm, Sigma-Aldrich, #G4649, #G8772, respectively, +2 beads 2.5–3.5 mm, VWR, #332124G) in 600  μL S.T.A.R. buffer (Roche Diagnostics, #03335208001) and mixing twice in TissueLyser II (Quiagen) at 25 Hz for five min. The suspensions were spun down (10 min, 13,000 rpm, 4 °C) before supernatants were collected and kept at 4 °C overnight. DNA was extracted by magnetic-particle processing using the mag midi kit (LGC, #40402) following the manufacturer's protocol. Eluates were aliquoted and stored at −20 °C. The DNA concentration was determined with Quant-iT™ 1X dsDNA HS Assay Kit (Invitrogen™, #Q33232) in Qubit® 2.0 Fluorometer (Invitrogen™, #Q32857) and integrity assessed by 1% agarose gel electrophoresis.

A 16S rRNA gene amplicon library was prepared through two-step amplicon PCR. The V3-V4 region of the 16S rRNA gene was amplified using 5x HOT FIREPol® Blend Master Mix Ready to Load (Solis BioDyne, #04-25-00115) and primers 341F (5′-CCTACGGGAGGCAGCAG-3′) and 806 R (5′-GGACTACCAGGGTATCTAAT-3′)^46^ on a GeneAmp® PCR System 9700 (Applied Biosystems). Amplicon integrity was evaluated by 1% agarose gel electrophoresis. PCR products were cleaned using BioMek4000 Automated Workstation (Beckman Coulter) by combining 10 μL of a magnetic particle suspension (0.1% Sera-Mag™ SpeedBead Carboxylate-Modified magnetic particle (Cytiva, #45152105050350) in a solution consisting of 1 M NaCl, 18% PEG-6000/PEG-8000, 10 mM Tris-HCl, 1 mM EDTA, and 0.05% Tween®20 in ultrapure water) with 10 μL of amplicon PCR product. The suspensions were incubated for five min, placed on a magnet for two min, and supernatant discarded. Beads were washed twice with 100 μL of 80% ethanol for 30 s and left to air dry for 15 min before removal from magnet, and 20 μL of nuclease-free water was added to the wells, mixed well, and incubated for two min. Finally, the samples were placed on the magnet for two min and eluates were collected.

Amplicons were indexed with unique dual Illumina index primers dispensed using epMotion 5070 robot (Eppendorf) and PCR run using FIREPol® Master Mix Ready to Load (Solis BioDyne, #04-12-00125) on GeneAmp® PCR System 9700 (Applied Biosystems). The products were validated with 1.5% agarose gel electrophoresis.

DNA concentration was determined using Quant-iT™ 1X dsDNA HS Working Solution (Invitrogen™, #Q33232) and relative fluorescence units (RFUs) measured in Varioskan LUX SkanIt Plate Reader. Samples with a wide range of RFUs were additionally measured in Qubit® 2.0 Fluorometer (Invitrogen™) to make a standard curve for estimating the DNA concentration of remaining samples. Samples were normalized during pooling of all the samples into one library using BioMek3000 (Beckman Coulter). The library was cleaned manually by incubating 150 µL pooled library with 0.8x volumes of 0.1% Sera-Mag™ SpeedBeads bead solution for five min. The suspension was placed on a magnet for two min and supernatant discarded. Beads were washed twice with 200 μL of 80% ethanol for 30 s, left to air dry for 15 min, removed from the magnet, and 40 μL of nuclease-free water was added, mixed well and incubated for two min. Finally, the suspension was placed on the magnet for two min and 35 μL of eluate was collected and stored at −20 °C. Concentration was measured by Qubit® 2.0 Fluorometer (Invitrogen™) as before, and the product was validated by 2% agarose gel electrophoresis. The library was subjected to paired-end (2 × 300 bp) sequencing in Illumina MiSeq v3 at the Norwegian Sequencing Center (UiO, Oslo, Norway) as previously described.^47^

Twenty DNA samples from week five of experiment 1 (before DSS treatment; *n* = 5 per group) were prepared for shotgun metagenome sequencing by the Norwegian Sequencing Center (UiO, Oslo, Norway) by Illumina DNA Prep (Tagmentation), following the manufacturer's protocol. Paired-end (2 × 150 bp) sequencing was performed with Illumina NovaSeq 6000 Sequencing System (1/4 S4 flow cell).

### Microbial community analyzes

The final 16S rRNA gene (V3–V4) amplicon dataset included 15,603,041 high-quality and chimera-checked sequences (5,102–125,247 per sample), representing 3298 amplicon sequence variants (ASVs). Raw sequence reads were demultiplexed and adapters and primers removed using midiv v2.2.0 (available at https://github.com/larssnip/midiv/blob/master/R/demultiplex.R) in the R programming environment (v4.3.1). ASVs were determined with DADA2 v1.30.0.^48^ The 3′ end of forward and reverse reads were trimmed 25 and 70 bp, respectively, followed by quality filtering at maxEE = 2.53 (forward) and 4.22 (reverse), to prevent analysis of regions with low-quality base calling. Taxonomy of the ASVs was assigned with SINTAX (available at https://www.drive5.com/usearch/manual/sintax_algo.html)^49^ using the species representative 16S from the GTDB database, release 220 (available at https://gtdb.ecogenomic.org/).^50^ Taxonomy for ASVs with a confidence score of > 0.8 were included further, while taxa with lower scores were assigned to “unclassified”.

Raw paired-end reads from shotgun sequencing were filtered and trimmed using BBDuk v39.06 (K = 23, hdist = 1; available at https://jgi.doe.gov/data-and-tools/software-tools/bbtools/), where 3′ end was trimmed of bases with Phred quality score Q < 20, and reads with average quality of Q < 20 or shorter than 30 bp were discarded. Read pairs were decontaminated of host genome (GRCm39, NCBI RefSeq GCF_000001635.27) using Bowtie 2 v2.5.3^51^ and SAMtools v1.19.2.^52^ Contigs were assembled sample-wise with metaSPAdes v3.15.5^53^ and binned using MetaBAT2 v2.15,^54^ MaxBin2 v2.2.7,^55^ and CONCOCT v1.1.0^56^ followed by ensemble binning using DAS_Tool v1.1.7.^57^ Finally, bins from all samples were given quality assessment and filtering using CheckM2 v1.0.1^58^ followed by a dereplication with dRep v3.4.5^59^ to obtain a final set of metagenome assembled genomes (MAGs) from all samples collectively. Requirements for a bin to qualify as a MAG: minimum completeness 75%, maximum contamination 25%. Taxonomy of the bins was assigned using GTDB release 2.20 and the tool GTDB-Tk v2.4.0.^60^

Raw sequence files and MAGs are deposited in the Sequence Read Archive and are available under accession number PRJNA1320514.

### Data processing and statistical analyzes

Raw quantification cycle (Cq) values obtained from RT‒qPCR of liver RNA samples were adjusted for variability between runs by calculating the genewise difference in the mean Cq for the IRC on plates 1 and 2 and adjusting all Cq values from plate 2 accordingly. In cases where reactions had no signal, Cq values were imputed to the maximum number of cycles to reduce bias from sample exclusion (liver: 40; mucosa: 30). Fold-change (FC) values were obtained using the Livak (FC = 2^−ΔΔCq^) method^61^ using the average of the two housekeeping genes *Tbp* and *Gapdh* and group-matched non-DSS-treated animals as the reference group. See **Supplementary Table S5** for Cq values and all results from statistical analyzes of mucosa gene expression data.

The 16S rRNA gene amplicon dataset was filtered by excluding samples with a total read count below 10,000 (one sample) followed by removing ASVs with zero counts across all the samples. ASV read counts were normalized to account for differences in sequencing depth by sample-wise division of the read count for each ASV by the sum of read counts in a given sample, multiplying by the lowest total read count number in the dataset (10,188), and rounding up to the nearest integer. For each subset of data (experiment and time point), only ASVs with relative abundance >0.25% in at least one sample were kept for statistical testing.

Microbial communities were analyzed in the R programming environment using phyloseq (v1.48.0) and vegan (v2.6-6.1). *The α*-diversity was estimated using Shannon effective index (exp(Shannon)). *β*-diversity was assessed using generalized UniFrac distances based on a GTDB-derived taxonomic tree. The tree topology reflects genome-based phylogenetic relationships defined by the GTDB, while branch lengths were set uniformly. The UniFrac distances, therefore, reflect shared taxonomic lineage depth, but since the GTDB taxonomy is based on phylogeny this is a good proxy for evolutionary divergence between taxa. Group differences in *β*-diversity were addressed using permutational multivariate analysis of variance (PERMANOVA) and tested for homogeneity of group dispersions (PERMDISP).

Associations between genus abundances and housing condition and/or diet were analyzed by a compound Poisson linear model (MaAsLin2 v1.18.0, available from https://www.bioconductor.org/packages/release/bioc/html/Maaslin2.html). *P*-values were FDR-adjusted, and q-values < 0.05 were considered significant. Input data consisted of filtered, normalized relative abundances of ASVs, and therefore, no further normalization or transformation was applied. For experiment 1, minimum prevalence and minimum abundance were set to 0.01 and 0.1, respectively, and a minimum prevalence of 0.08 was used for experiment 2.

The functional annotation of predicted genes in the microbial genomes (MAGs) was performed with the DRAM software v1.5.^62^ Relative abundance of each MAG per sample was calculated as the number of reads assigned to each MAG relative to the total number of reads in that sample. Relative abundance of CAZymes per sample was obtained from the sum of relative CAZyme abundance across MAGs in that sample, calculated as the product of the number of CAZyme hits in the MAG and its relative abundance in that sample.

All the statistical methods applied are specified in the figure legends. Sex‑dependent effects could not be assessed statistically due to single‑sex composition in one group at termination. Homogeneity of variance was evaluated with Levene's or Brown-Forsythe test for heteroscedasticity, and normality of residuals was tested by Shapiro–Wilk normality test and inspection of Q–Q-plots in GraphPad Prism 10 (v10.1.2) or R.

## Results

### Feralized laboratory mice were protected against DSS-induced colitis independent of the diet

**Figure 2.**
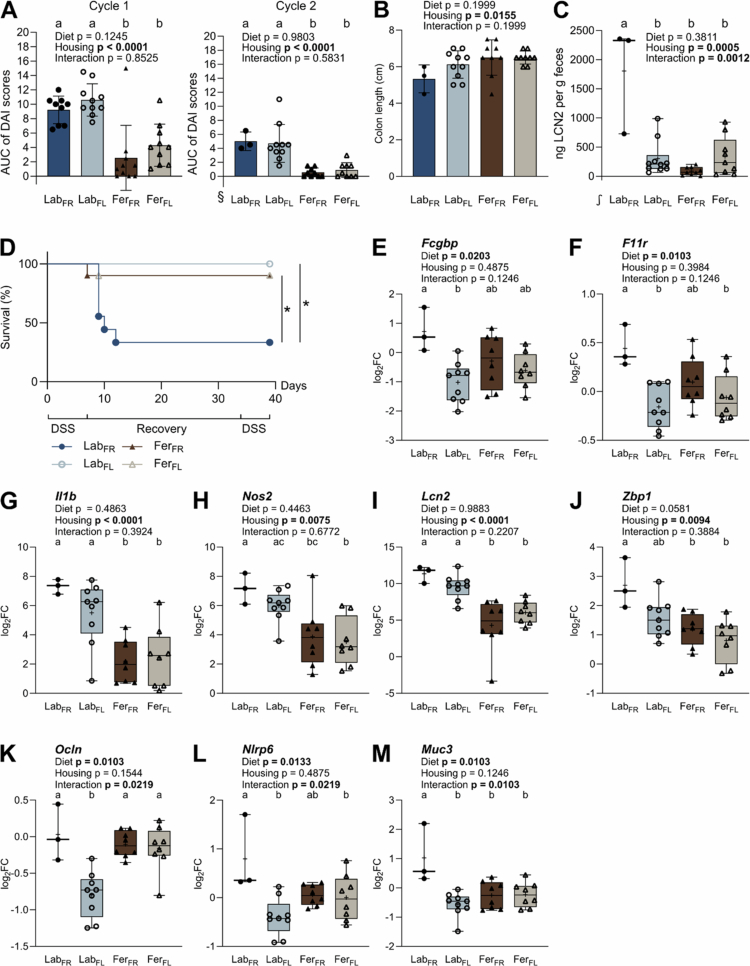
Clinical outcomes and gene expression in the colonic mucosa following DSS-induced colitis. (A) AUC of DAI scores given during cycle one (left) and two (right) of DSS-treatment. (B) Length of the colon in centimeters measured at the end of the experiment. Bar plots show individual mice (symbols), mean (bar), and SD (whiskers). Box plots show individual mice (symbols), median (line), mean (+), IQR (box), and 1.5 IQR/minimum to maximum (whiskers). (C) Fecal levels of Lipocalin-2 (LCN2) in ng per g feces measured at the end of the experiment. Levels in untreated animals in **Supplementary Figure S3**. (D) Kaplan‒Meier survival plot for the DSS treatment period until termination (40 d). *, adjusted *p*-value < 0.05 following pairwise Gehan-Breslow-Wilcoxon tests; *p* values adjusted with two-stage linear step-up procedure of Benjamini, Krieger and Yekutieli, Q: 5%. (E-M) Relative expression of barrier-related genes in colonic mucosa collected at the end of the experiment, presented as log2FC of DSS-treated animals relative to untreated animals. See **Supplementary Table S5** for additional information on statistics and abundance values. Dark blue/filled dots, Lab_FR_ (*n* = 3–9); light blue/open dots, Lab_FL_ (*n* = 9–10); brown/filled triangles, Fer_FR_ (*n* = 8–10); beige/open triangles, Fer_FL_ (*n* = 8–10). All except D show Benjamini–Hochberg adjusted *p* values for the effect of diet, housing condition, and interaction determined by two-way ANOVA. Letters designate significant (*p*-value ≤ 0.05) differences between groups following Tukey's multiple comparison testing. Statistics performed on transformed data: § square root; ∫, log10. DSS, dextran sulfate sodium; AUC, area under the curve; DAI, disease activity index; Lab, laboratory; Fer, feralized; FR, fiber rich; FL, fiber low.

Five weeks after exposure to feralization (Fer) or clean lab conditions (Lab) with or without fermentable dietary fibers (DFs), mice were challenged with 1% DSS in two cycles separated by a 16-d recovery period. Disease activity index (DAI) scores were lower in Fer mice than Lab mice in both cycles (Figure 2A and B), aligning in part with the fecal levels of colitis biomarker Lipocalin-2 (LCN2) measured at termination of the experiment (week nine; Figure 2C, untreated animals in **Supplementary Figure S2**). During the recovery period, six animals in Lab_FR_ had reached endpoints requiring euthanasia (weight loss > 20%), and one mouse had been previously euthanized for unrelated reasons (Figure 2D, **Supplementary Figure S1**). Two Fer mice (one FR and one FL) were euthanized following the first DSS cycle due to significant weight loss (>20%). As most remaining animals in these groups exhibited minimal symptoms and <5% weight loss, the euthanized mice were likely not representative of their respective groups.

Gene expression analysis of the colon mucosa revealed altered relative expression of nine gut-barrier-related genes, depending on the diet (*Fcgbp* and *F11r*, Figure 2E–F), housing condition (*Il1b*, *Nos2*, *Lcn2*, and *Zbp1*, Figure 2G–J), or both diet and the interaction between diet and housing (*Muc3*, *Nlrp6*, and *Ocln*, Figure 2K–M). Groupwise comparisons showed that overall, Lab_FR_ mice displayed the highest relative expression of these genes compared to the other groups. Both Fer groups had reduced expression of genes encoding pro-inflammatory cytokine IL-1β, LCN2-encoding *Lcn2*, and reactive oxygen species-producing NOS2/iNOS (trend for Fer_FR_ vs. Lab_FL_) compared to Lab. Both FL groups showed lower expression of mucus-structure-related *Fcgbp* (trend for Fer_FL_) and tight-junction protein-encoding *F11r*compared to Lab_FR_, while no differences between the two Fer groups were observed for these genes, suggesting no effect of the diet within the Fer groups. Finally, Lab_FR_ mice had higher expression of inflammasome *Nlrp6* (trend for Lab_FR_ vs. Fer_FR_) and transmembrane mucin-encoding *Muc3* compared to all other groups.

Relative expression of *Lcn2*, *Nos2*, *Il1b*, *Ocln*, and *Zbp1* correlated significantly with DAI (**Supplementary Figure S3**), suggesting the expression level of these genes was affected according to disease development. Taken together, these results show that feralizing laboratory mice mitigates clinical DSS-induced colitis symptoms and local inflammation in the colon compared to mice housed in a clean environment. The diet had greater impact on Lab mice, in which the FR diet exacerbated colitis severity and increased the expression of genes related to mucus, barrier junctions, and inflammation compared to the FL diet.

### Feralized mice exhibited less pronounced systemic inflammation compared to laboratory mice

**Figure 3.**
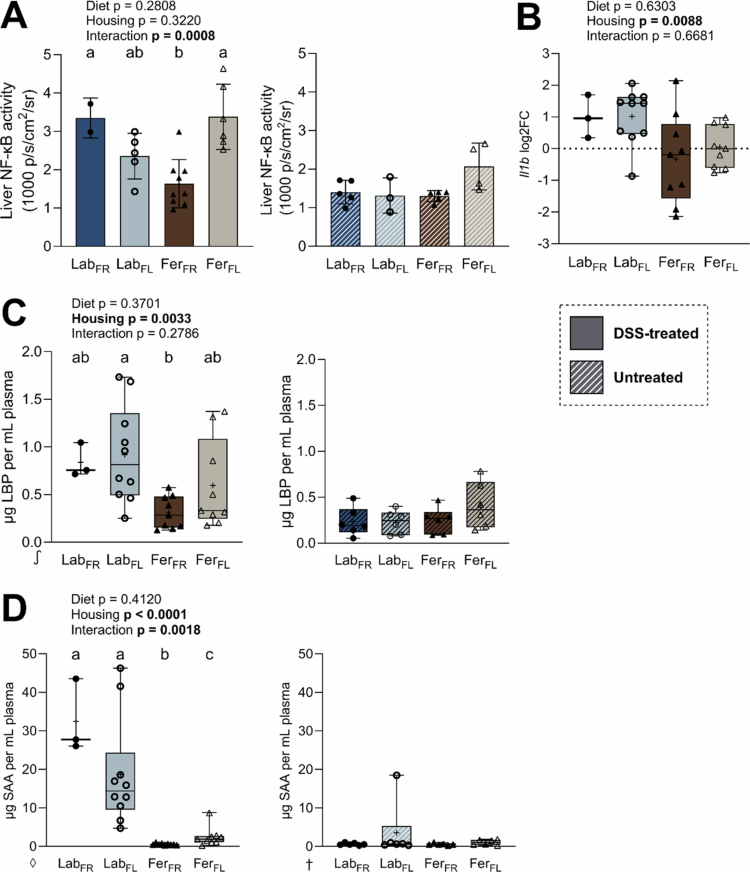
Measurements of NF-κB activity and systemic inflammation markers. (A) NF-κB activity in the liver of DSS-treated (left) and untreated (right) mice as average radiance of emitted bioluminescence as 1000 photons per second per area per steradian. (B) Relative expression of *Il1b* in the livers of DSS-treated animals relative to untreated animals as log2 of fold change. (C) μg lipopolysaccharide-binding protein (LBP) per mL plasma in DSS-treated (left) and untreated (right) mice measured after the first cycle of DSS treatment. (D) μg serum amyloid A (SAA) per mL plasma in DSS-treated (left) and untreated (right) mice measured after the second cycle of DSS treatment. Box plots show individual mice (dots/triangles), median (line), mean (+), IQR (box), and minimum to maximum (whiskers). Bar plots show individual mice (dots/triangles), mean (bar) and SD (whiskers). Dark blue/filled dots, Lab_FR_ (*n* = 2–3); light blue/open dots, Lab_FL_ (*n* = 5–10); brown/filled triangles, Fer_FR_ (*n* = 9); beige/open triangles, Fer_FL_ (*n* = 6–9). All untreated groups: *n* = 3–6. The *p* values for the effect of diet, housing condition, and interaction determined by two-way ANOVA are presented, while letters designate significant differences between groups following Tukey's multiple comparison testing (adjusted *p*-value ≤ 0.05). Statistics performed on ◊, log_2_; or ∫, log_10_ transformed data. DSS, dextran sulfate sodium; Lab, laboratory; Fer, feralized; FR, fiber rich; FL, fiber low; FC, fold change; NF-κB, nuclear factor kappa-light-chain-enhancer of activated B cells.

To evaluate whether the colon inflammation triggered effects beyond the local site, NF-κB activity in livers of DSS-treated B6-NF-κB^Luc^ reporter mice was examined by *ex vivo* imaging. Measurements of NF-κB activity in excised livers revealed significantly lower activity in the Fer_FR_ mice compared to the other groups (Figure 3A, left panel), whereas no significant differences were observed in untreated animals (Figure 3A, right panel). Relative expression of *Il1b* mRNA in the liver was also lower in Fer compared to Lab animals, but was not affected by the diets (Figure 3B). We further measured plasma levels of liver-derived acute-phase proteins lipopolysaccharide-binding protein (LBP) and serum amyloid A (SAA). LBP is regarded as a proxy of LPS leaking from the gut, and SAA is greatly elevated during systemic inflammation.^63^ After the first week of DSS treatment, LBP was significantly lower in Fer mice compared to Lab mice (Figure 3C, left panel). A similar pattern was observed for SAA at termination (Figure 3D, left panel). Fer_FR_ mice equaled untreated animals in both cases (Figure 3C+D, right panels). Collectively, these results indicate that feralization confers protection against induction of systemic inflammation following DSS-induced colitis, especially when combined with an FR diet.

### Both feralization and diet drove marked shifts in fecal microbiota compositions

**Figure 4.**
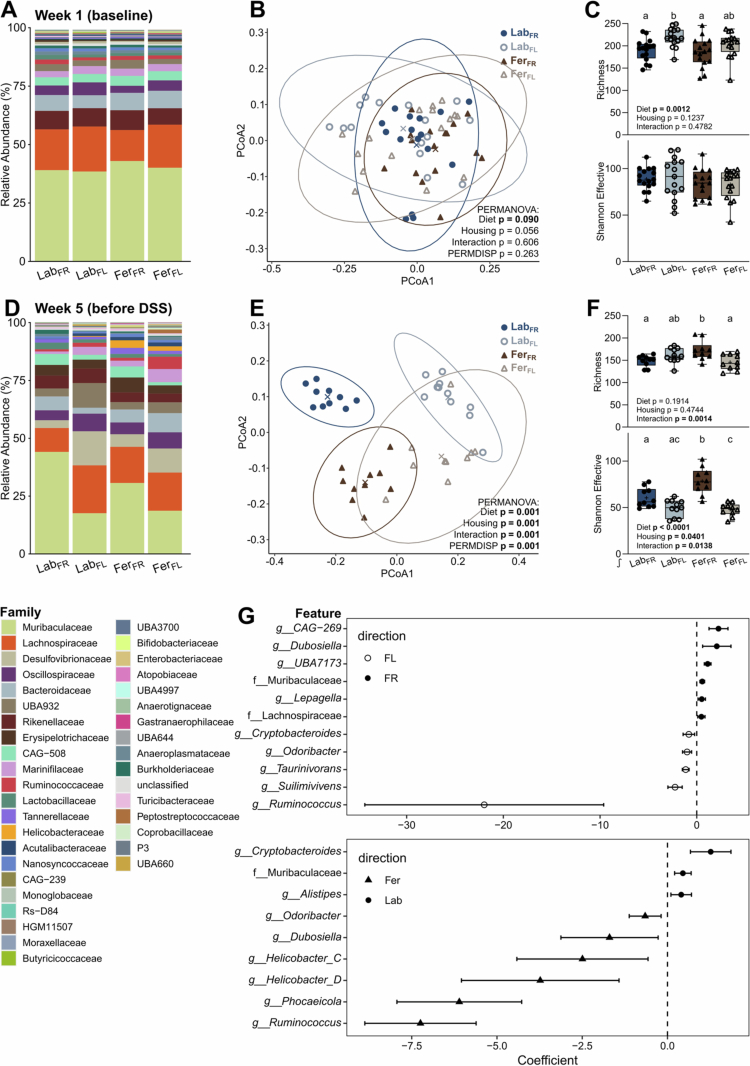
Fecal microbiota profiles determined by 16S rRNA gene amplicon sequencing at baseline and after five weeks of feralization in a farmyard-like habitat and dietary fiber intervention. (A + D) Taxonomic binning at family level, presented as relative abundance across groups at baseline and week five, respectively. (B + E) Principal coordinates analysis (PCoA) plot of fecal microbiota profiles (generalized UniFrac distances) at baseline and week five, respectively. X presents the centroids, and the ellipses embrace the 95% confidence level. Significant separation of groups was determined by permutational analysis of variance (PERMANOVA) and dispersion of groups determined by permutation of dispersions (PERMDISP) using the spatial median. Presented are *p*-values of main effects (housing and diet) and their interaction (significant *p*-value ≤ 0.05). (C + F) Richness (upper panel) and Shannon effective counts (lower panel) at baseline and week five, respectively, presented as box plots show median (line), mean (+), IQR (box) and minimum to maximum (whiskers). Dark blue/filled dots, Lab_FR_ (*n* = 10–16); light blue/open dots, Lab_FL_ (*n* = 11–15); brown/filled triangles, Fer_FR_ (*n* = 10–16); beige/open triangles, Fer_FL_ (*n* = 10–16). *p* values for the effect of diet, housing condition, and interaction determined by two-way ANOVA are given, and letters designate significant (adjusted *p*-value ≤ 0.05) differences between groups following Tukey multiple comparison testing. ∫, statistics performed on log_10_-transformed data. (G) Forest plot of taxa significantly associated with diet (upper panel; open dots and negative coefficient, FL; filled dots and positive coefficient, FR) and housing condition (lower panel; triangle and negative coefficient, Fer; dot and positive coefficient, Lab) as determined by MaAsLin2. The coefficient represents the estimated linear effect size of relative genus abundance with 95% confidence intervals (horizontal lines). Lab, laboratory; Fer, feralized; FR, fiber rich; FL, fiber low.

We next evaluated the extent to which feralization and diet intervention affected microbiota composition to gain insight into the gut community that preceded the DSS-induced colitis. Fecal microbiota was analyzed by 16S rRNA gene amplicon sequencing at baseline (before exposure to experimental housing conditions and diets) and five weeks after their introduction (immediately preceding DSS treatment). At baseline, all groups displayed comparable microbiota profiles (Figure 4A–C), while they differed significantly after five weeks of feralization and/or diet intervention (Figure 4D–F). Both feralization and diet induced distinct compositional and phylogenetic shifts in overall microbiota structure (Figure 4E). Among the four groups, Fer_FR_ displayed the highest richness and effective Shannon counts, indicating a higher level of *α*-diversity compared to the other groups (Figure 4F). Multivariable association analysis with MaAsLin2 to identify differentially abundant (q < 0.05) genera revealed significant fiber-related effects (Figure 4G, upper panel). Mice fed the FR diet exhibited enrichment of *Dubosiella* (Erysipelotrichaceae), *Lepagella* (Muribaculaceae), *CAG-269* (CAG-508), *UBA7173* (Muribaculaceae), and two unclassified genera within Muribaculaceae and Lachnospiraceae, whereas mice fed the FL diet were associated with higher abundance of *Cryptobacteroides* (UBA932), *Odoribacter* (Marinifilaceae), *Taurinivorans* (Desulfovibrionaceae), *Suilimivivens* (Lachnospiraceae), and *Ruminococcus* (Ruminococcaceae).

Moreover, we detected housing-associated shifts in microbial abundance, such as an enrichment of *Helicobacter* in feralized mice (Figure 4G, lower panel). *Helicobacter spp.* readily colonize naturalized mice, as observed previously,^7^^,^^16^^,^^21^ and was detected in 19/20 Fer mice. In contrast, only one Lab mouse had detectable levels of this taxon. Other genera displaying higher abundance in Fer mice included *Odoribacter* (Marinifilaceae), *Dubosiella* (Erysipelotrichaceae), *Phocaeicola* (Bacteroidaceae), and *Ruminococcus* (Ruminococcaceae), whereas *Cryptobacteroides* (UBA932), *Alistipes* (Rikenellaceae), and the unclassified Muribaculaceae were more abundant in Lab groups (Figure 4G, lower panel). In summary, the fecal microbial composition was altered depending on both feralization and diet, shaping different communities preceding the induction of colitis.

As a high-fiber diet is associated with increased SCFA production and affects both the microbiota, epithelial barrier function, and immunity,^64^ we measured cecal levels of SCFAs. We detected no differences between groups among the DSS-treated animals (**Supplementary Figure S4**, left panels). However, untreated Lab_FR_ mice showed higher SCFA levels compared to Lab_FL_, while no significant differences were observed between Fer mice (**Supplementary Figure S4**, right panels). Hence, the protective mechanisms against colitis elicited by feralization as observed in our current study, do not appear to be explained by altered SCFA levels prior to DSS treatment.

### Fecal microbiota transfer of a feralized microbiota reproduced protection against DSS

**Figure 5.**
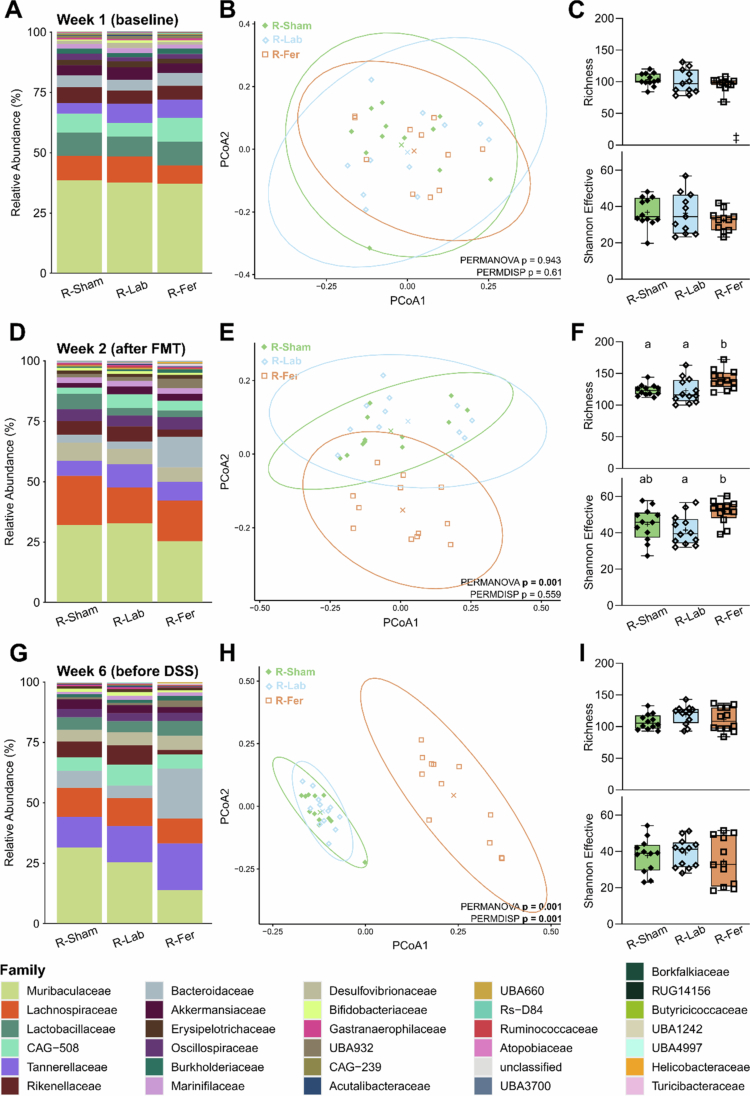
Fecal microbiota profiles determined by 16S rRNA gene amplicon sequencing at baseline and after fecal microbiota transfer. (A–C) Microbial composition measured at baseline, (D–F) one week after FMT, and (G–I) five weeks after FMT. (A, D, G) Taxonomic binning at family level, presented as relative abundance across groups. (B, E, H) Principal coordinates analysis (PCoA) plot of fecal microbiota profiles (generalized UniFrac distances). X presents the centroids and ellipses embrace the 95% confidence level. Significant separation of groups was determined by permutational analysis of variance (PERMANOVA) and dispersion of groups determined by permutation of dispersions (PERMDISP) using the spatial median (significant *p*-value ≤ 0.05). (C, F, I) Richness (upper panel) and Shannon effective counts (lower panel) presented as box plots show median (line), mean (+), IQR (box) and minimum to maximum (whiskers). Green/filled diamonds, R-Sham (*n* = 12); blue/open diamonds, R-Lab (*n* = 12); orange/open square, R-Fer (*n* = 12). Letters designate significant differences between groups determined by one-way ANOVA following Tukey multiple comparison test (adjusted *p*-value ≤ 0.05). ‡, Brown-Forsythe ANOVA. R, recipient; Lab, laboratory; Fer, feralized; FMT, fecal microbiota transfer.

**Figure 6.**
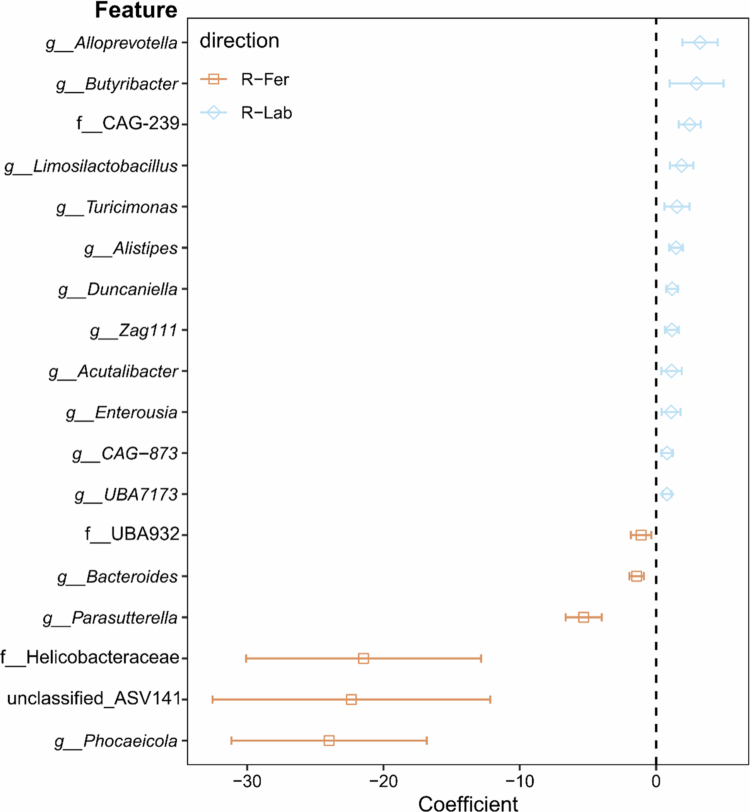
Differentially abundant taxa in FMT recipients determined by MaAsLin2. Analysis of 16S rRNA gene amplicon sequencing data binned at genus level in fecal microbiota from week 6 of experiment 2. Forest plot of taxa (features) significantly associated with R-Lab (blue open diamonds, positive coefficient) and R-Fer (orange squares, negative coefficient). The coefficient represents the estimated linear effect size of relative genus abundance with 95% confidence intervals (horizontal lines).

The Fer_FR_ and Lab_FR_ groups presented the most contrasting fecal microbial communities and diverging responses to DSS treatment. Thus, we hypothesized that the feralized microbiota was a key factor underlying the observed protection against colitis in Fer_FR_. We investigated whether transferring microbiota from these two groups of mice would confer similar effects in recipient mice upon DSS-induced colitis. Fecal microbiota was transferred from either Fer or Lab donors fed an FR diet to three-week-old B6 pups (R-Fer and R-Lab, respectively), and fecal microbiota in recipients was assessed at baseline (Figure 5A–C) and one and five weeks after FMT to confirm successful and sustained engraftment of donor microbiota (Figure 5D–F and G–I, respectively). The resulting microbial profiles revealed a persistent shift in recipient mice of a feralized microbiota (R-Fer) (Figure 5B, E A, and H). In contrast, the microbiota profiles of recipients of the Lab microbiota (R-Lab) remained similar to vehicle-treated B6 mice (R-Sham). Only a transient increase in richness and Shannon Effective index in R-Fer was observed (Figure 5C, F, and I). MaAsLin2 analysis was again performed to identify genera differentially abundant (q < 0.05) between R-Fer and R-Lab (Figure 6). Several taxa were significantly associated with R-Fer, including *Bacteroides* (Bacteroidaceae), *Parasutterella* (Burkholderiaceae), *Phocaeicola* (Bacteroidaceae), two unclassified genera belonging to families Helicobacteraceae and UBA932, and one unclassified genus represented by a single ASV (ASV141). R-Lab was significantly associated with genera like *Duncaniella* (Muribaculaceae), *Alistipes* (Rikenellaceae), *Alloprevotella* (Lachnospiraceae), *Butyribacter* (Lachnospiraceae), and *Turicimonas* (Burkholderiaceae).

**Figure 7.**
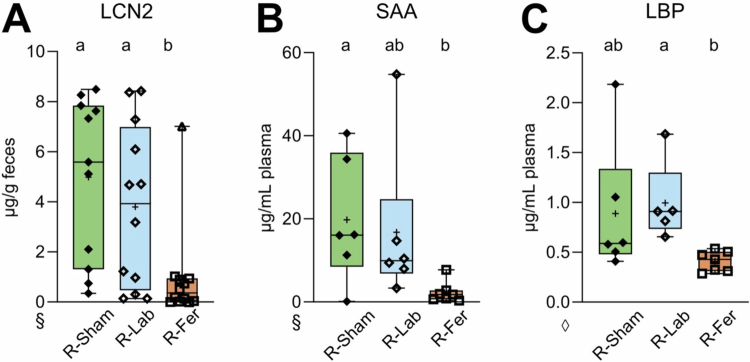
Levels of plasma and fecal inflammatory biomarkers following DSS-induced colitis in FMT recipients. (A) Fecal levels of Lipocalin-2 (LCN2), (B) plasma levels of Serum amyloid A (SAA) and (C) lipopolysaccharide-binding protein (LBP) were determined by ELISA. Box plots show median (line), mean (+), IQR (box) and minimum to maximum (whiskers). Green/filled diamonds, R-Sham (*n* = 6–11); blue/open diamonds, R-Lab (*n* = 5–12); orange/open square, R-Fer (*n* = 7–12). Letters designate significant (adjusted *p*-value ≤ 0.05) differences between groups determined by one-way ANOVA following Tukey multiple comparison test. Statistics performed using ◊, log2; or §, square root transformed data. R, recipient; Lab, laboratory; Fer, feralized; FMT, fecal microbiota transfer.

Importantly, we reproduced the protective effects of a feralized microbiota adapted to an FR diet observed in experiment 1, as shown by the measurements of biomarkers of inflammation after colitis induction. Significantly lower levels of fecal LCN2 and plasma LBP (Figure 7A+C), and a trend for plasma SAA (*p* = 0.056, Figure 7B) were found in R-Fer compared to R-Lab. As in experiment 1, cecal SCFA levels after DSS showed no differences between groups (**Supplementary Figure S5**). These findings confirm that the protective effect conferred by feralization against DSS-induced local and systemic inflammation was through microbial influence.

### Bacterial genomes revealed a shift in the genomic potential of the gut microbiota largely dependent on diet

**Figure 8.**
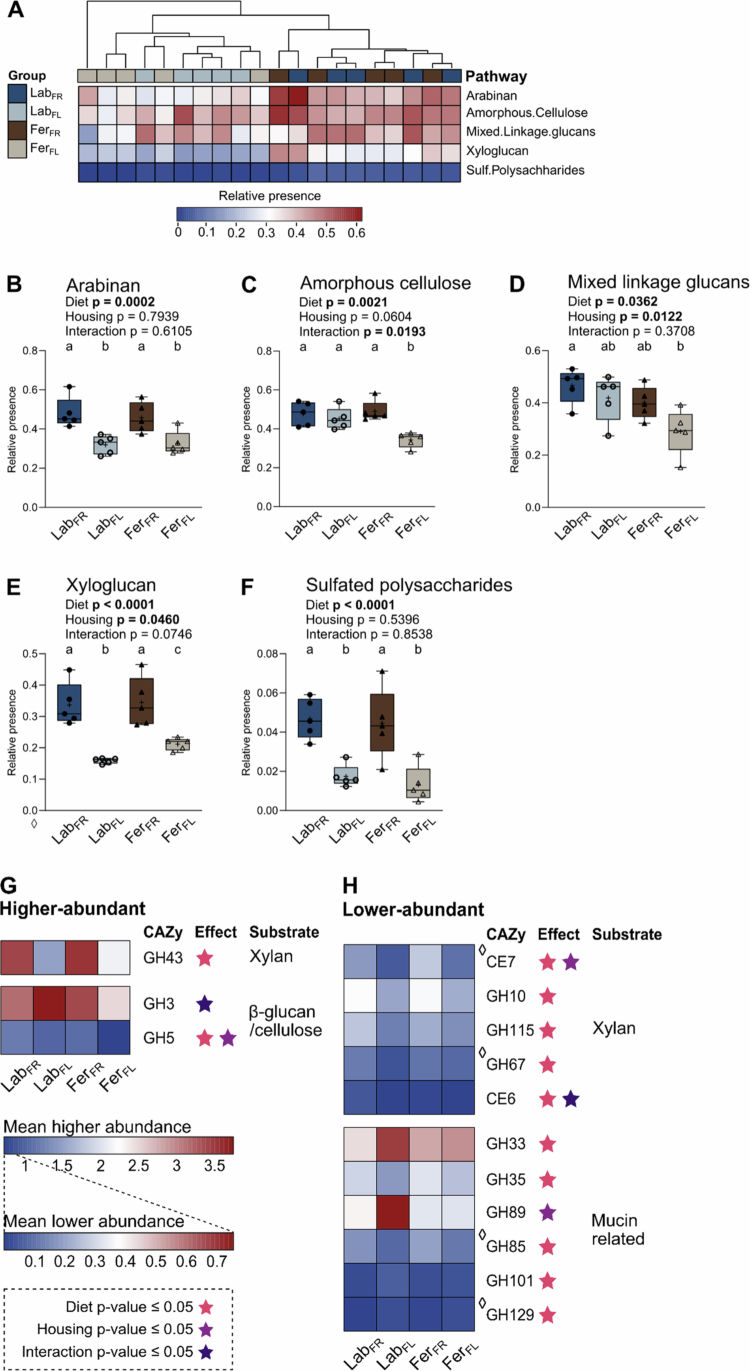
Relative presence of pathways and relative abundance of predicted CAZyme genes involved in xylan, *β*-glucan, and mucin degradation in the fecal microbiome. Relative presence of CAZy pathways presented as (A) a heatmap and (B–F) boxplots for each pathway in the four experimental groups. Samples in heatmap were plotted by hierarchical clustering using average linkage of Canberra distances. Relative presence is the dot product of relative abundance of each MAG and the presence of each pathway within them. Box plots show median (line), mean (+), IQR (box) and minimum to maximum (whiskers). Dark blue/filled dots, Lab_FR_; light blue/open dots, Lab_FL_; brown/filled triangles, Fer_FR_; beige/open triangles, Fer_FL_. *n* = 5 for all groups. Heatmap presenting the mean relative abundance of (G) higher and (H) lower abundant CAZy families (dot product of relative abundance and gene counts within MAGs) that were significantly affected by diet, housing condition, or their interaction, determined by two-way ANOVA (Benjamini‒Hochberg adjusted *p* values ≤ 0.05). Only significant CAZymes are shown, see complete list and individual values in **Supplementary Table S6**. The *p*-values for the effect of diet, housing condition, and interaction determined by two-way ANOVA are given, and letters designate significant differences between groups following Tukey multiple comparison testing (adjusted *p*-value ≤ 0.05). ◊, statistics performed on log_2_-transformed data. Lab, laboratory; Fer, feralized; FR, fiber rich; FL, fiber low; CAZy, carbohydrate-active enzymes; GH, glycoside hydrolase; CE, carbohydrate esterases.

We found that Fer_FR_, but not Lab_FR_, was protected against DSS-induced colitis, a trait also transferable by FMT, and therefore proceeded to explore whether the microbiota composition of these mice differed with respect to their genomic potential for degrading DFs and mucins. We investigated 317 metagenome-assembled genomes (MAGs, **Supplementary Figure S6**) constructed from shotgun sequencing of fecal DNA collected from all four groups of experiment 1 at week five, prior to DSS treatment. Functional annotation of predicted genes within MAGs revealed distinct differences in the genomic potential to degrade complex carbohydrates between the gut microbiota of mice fed an FR versus an FL diet (Figure 8A). Specifically, the presence of groups of genes encoding enzymes needed for specific metabolic pathways was primarily influenced by diet (Figure 8A, **Supplementary Table S6**). Irrespective of housing condition, the microbiomes of FR mice were enriched in metabolic pathways related to the degradation of arabinan, xyloglucan, and sulfated polysaccharides (Figure 8B–F). A somewhat higher potential for utilizing MLG was observed in Lab compared to Fer mice, as well as FR compared to FL mice within each housing condition. Enriching the diets with DFs thus appeared to shift the genomic potential of the microbiome toward a greater ability to degrade complex carbohydrates, suggesting an adaptation of the gut microbiota to available substrates. This appeared predominantly independent of feralization.

Given the different substrates provided by the FR and FL diets, we further assessed whether the experimental groups were differentially enriched in specific carbohydrate-active enzymes (CAZymes) relevant to these, which could indicate differential utilization of DFs between the groups. Additionally, glycoside hydrolases (GHs) relevant to mucin degradation (the mucin core GH-ome and related GHs)^65^ were included in this analysis. First, the mean relative abundance of MAGs differed between groups and largely aligned with profiles observed through 16S rRNA gene amplicon sequencing at family level (**Supplementary Figure S7A**). Second, assessing the number of predicted CAZyme genes in the top 20 most abundant MAGs in each group revealed some that harbored particularly high numbers of specific CAZymes (**Supplementary Figure S7B-E**). For example, MAGs assigned to *Bacteroies muris, Phocaeicola vulgatus*, *Duncaniella muricolitica*, *Bacteroides acidifaciens, Lepagella muris*, and *Muribaculum gordoncarteri* possessed extensive CAZyme repertoires, with the CAZymes of interest comprising a substantial proportion of their total enzymatic potential. There were also several unknown species in Muribaculaceae (*UBA7173* spp., *UBA3263* spp., and *JAGBWK01 sp947174275*) and *Alistipes* spp. that presented with several predicted mucin core GH-ome genes. Finally, combining these data allowed for visualizing a CAZyme abundance in the mouse groups, revealing that several CAZy families with functions involved in degrading arabinoxylan, *β*-glucans/cellulose, and mucins were significantly different between the groups depending almost exclusively on their diet (Figure 8G+H, **Supplementary Table S6**). As expected, bacterial genomes from FR mice were enriched in genes encoding arabinoxylan-degrading enzymes (GH43, GH10, GH115, GH67, CE6, CE7) compared to FL mice. For *β*-glucan/cellulose degradation, higher levels of GH5 were observed in FR than FL mice (and significantly lower in Fer_FL_ than all other groups), whereas GH3 was significantly higher in Lab_FL_ than Fer_FL_. Lab_FL_ mice were found to be particularly rich in CAZymes related to mucin degradation (GH89, GH33, GH129, GH101). Both FL groups showed higher levels of GH129 compared to Fer_FR_, while levels of GH35 and GH85 were higher in the FR groups. Taken together, the collective potential for degrading the carbohydrates in the diets suggests that the gut microbiome of FR mice, regardless of housing condition, became enriched in genes encoding CAZymes related to xylan-degradation, whereas the FL-adapted microbiome shifted towards a more mucin-degrading gene profile.

Since the microbiomes of Fer_FR_ and Lab_FR_showed similar genomic potential in utilizing the DFs, we next investigated whether the species contributing with these genes were different between groups. We compared which MAGs contributed most to the total abundance of each CAZy family within each group. For CAZy families present at comparable levels in both Lab and Fer mice, the most dominant contributor species differed in most cases (**Supplementary Table S7**). Out of the 27 CAZymes analyzed, CE2 was the only one where the greatest contributor was the same for all groups (*Alistipes* sp002358415). The top contributor differed between Lab and Fer mice in 18 (FR diet) and 21 (FL diet) CAZymes (**Supplementary Table S7**). For example, members of the Muribaculaceae family were major contributors to several CAZymes in both FR groups, but the dominating species differed between Lab_FR_ (*Lepagella muris*) and Fer_FR_ (*Muribaculum gordoncarteri*). Several top contributors were shared among both FL groups, including *Akkermansia muciniphila*, *Alistipes* spp., and *Acetatifactor* spp.

Beyond carbohydrate degradation, several bacterial metabolites associated with dietary fiber fermentation are relevant to inflammatory processes in the host, including deconjugated and secondary bile acids, tryptophan derivatives, hydrogen sulfide, and polyamines.^66^ We therefore examined the relative abundance of genes encoding key enzymes involved in their production as well as those involved in SCFA production. Of the 19 genes that were annotated, significant differences between the groups were found in ten of them (**Supplementary Figure S8**, **Supplementary Table S8**). Like the CAZymes, the most notable differences in these additional genes were driven by diet. FL groups showed greater potential for bile acid deconjugation (bile salt hydrolase, *bsh*) and hydrogen sulfide generation (dissimilatory sulfite reductase, *dsrAB*) compared to the FR groups. For the SCFAs, the relative gene abundance of *buk*/*ptb* and *bcoat,* both critical for butyrate production, were significantly higher in both FL groups compared to the FR groups, contrasting our observation of higher levels of cecal butyrate in untreated Lab_FR_ mice. The potential for acetate production, indicated by *pta* and *ackA* was higher in FR compared to FL groups, whereas the opposite was observed for *pduP* (propionate production).

Collectively, these observations suggest that the metabolic potential of the microbiome is mainly influenced by diet. While the feralizing environment reshapes the microbial composition, it seems these functional capacities are maintained across housing conditions.

## Discussion

We have previously shown that feralization of laboratory mice alters gut microbiota and gut barrier properties,^15^^,^^16^ and confers protection against colorectal carcinogenesis in both spontaneous and chemically induced models.^14^ The current study aimed at further evaluating the effect of feralization on gut inflammation by chemically inducing colitis with DSS. Given the association between a high intake of DFs and a reduced risk of several gut-related diseases, such as CD and UC, the mouse diets were either deprived of or enriched with fermentable DFs to assess their role in conjunction with feralization. We found that Fer mice overall were protected against colitis compared to Lab mice. While DFs provided slightly more protection in Fer mice, the opposite was observed in fiber-fed Lab mice, as DSS treatment was detrimental to these mice. These effects were reproduced in the FMT experiment, confirming that they were microbiota-driven.

Fer mice further appeared to be protected against systemic inflammation compared to Lab mice. Systemic inflammation in Lab mice likely resulted from gut-derived pro-inflammatory components, such as LPS, indicated by higher plasma LBP levels—a consequence of pronounced gut inflammation. This barrier disruption was also indicated by the elevated expression of genes in Lab_FR_ colonic mucosa, including inflammasome-encoding *Zbp1* (Z-DNA-binding protein 1), iNOS-encoding *Nos2*, and LCN2-encoding *Lcn2.* Elevated *Zbp1* expression has been shown to be associated with UC^67^ and to regulate inflammation-driven cell death,^68^ and *Nos2* and *Lcn2* have been identified as key upregulated genes in the colon of both UC and CD patients as well as mouse models of colitis.^69^ We have previously observed that genes encoding proteins central to maintaining and protecting the gut barrier show higher expression in colonic epithelial cells from naturalized mice,^16^ suggestive of activated responses to the gut microbiota. Higher expression of these genes may be indicative of an alteration of the gut barrier, which in turn could potentially provide resilience to barrier insults. It is important to note that the higher‑than‑anticipated attrition in the Lab_FR_ group resulted in smaller sample sizes for these analyzes, which should be taken into account when interpreting the results from the first experiment presented herein. Nonetheless, the replication of these findings in the second experiment, which included a larger sample size, supports the conclusions drawn.

Since Lab and Fer mice showed such contrasting disease outcomes, we investigated the microbial community in the gut immediately prior to DSS treatment to assess whether it could help explain the different disease outcomes. Lab mice overall had lower *α*-diversity measures than Fer_FR_ mice, highlighting that they harbor fewer species that were also less evenly distributed, considered unfavorable to gut health.^70^ However, this was not the case for Fer_FL_ mice, who displayed richness at the same level as the Lab groups, and the lowest Shannon effective numbers compared to the other groups, while still being largely protected against colitis. The fact that DFs significantly impacted Shannon effective numbers, causing an increase in FR groups, indicates that the DFs help rebalance the microbiome, evening out the species distribution. Our results indicate two things: Firstly, the DFs contributed to the colonization and even distribution of microbes from the feralizing environment, agreeing with a study by Seedorf et al. demonstrating that a microbe's ability to colonize correlated with its DF-utilizing properties.^36^ Secondly, while it seems to be beneficial with a higher *α*-diversity, a selection of specific microbial taxa may be crucial in conferring the effects observed in naturalized mice. In line with the second statement, R-Fer mice displayed only a transient increase in *α*-diversity measures. Similar observations have been made previously,^14^ supporting the notion that the mouse gut may not be a realized niche for some of the microbes present in the feralizing environment, and are likely outcompeted by inherent mouse gut microbes over time.^36^

Considering that a high intake of DFs is generally accepted to reduce risk of gut-related diseases,^71^ the pronounced disease burden of Lab_FR_ may seem unexpected. However, several mouse studies show variable effects of administering different DFs. Arifuzzaman et al. recently reported poor recovery in mice fed a purified diet (similar to AIN-93M, the FL diet used herein) supplemented with the highly fermentable fiber inulin following DSS-induced colitis.^72^ Their results suggested that inulin supplementation inhibited tissue repair in a microbiota-dependent manner, in which inflammation was promoted by microbiota-derived bile acids through the activation of inflammatory innate lymphoid cells (ILC2). Moreover, Miles et al. also found that adding inulin to an AIN-93M-like purified diet exacerbated colitis in mice, causing severe systemic inflammation that required euthanasia of many animals.^73^ Interestingly, this effect was not observed when inulin was added to regular chow, indicating that effects of DFs are context dependent. In line with these observations, several other studies find that susceptibility to colitis is DF-dependent: Pectin (particularly with high side-chain contents) ameliorates colitis, possibly in a microbiota-independent manner;^74‐76^ cellulose confers more protection than low or no cellulose (i.e., no fiber);^77^^,^^78^ guar gum and resistant starch protect more than cellulose or no fiber;^79^ soluble oat fiber (i.e., *β*-glucan) ameliorates colitis;^80^ and rhamnogalactorunan protects compared to insoluble fiber.^81^

Moreover, recent works by Caruso et al. showed that double knockout B6 mice (Nod2^-/-^ Cybb-^/-^) with a Taconic flora (Tac-DKO) develop spontaneous CD-like colitis, whereas these DKO mice with a Jackson flora do not.^82^ The same research group further identified that depriving Tac-DKO mice of DFs protected against CD-like colitis compared to regular chow.^83^ Worth noting is that the fiber-deficient diet used was a purified diet, whereas regular chow was not. This provides an interesting parallel to our findings: the specific combination of the gut microbiome and diet determines disease outcome. This was also recently shown by Bonazzi et al. ^84^ in which human microbiotas responded individually to inulin, psyllium, and cellulose *in vitro*. Furthermore, mice receiving FMT from these different human donors and fed different DFs also displayed different outcomes of DSS-colitis that depended on both donor and DF. Along with our results, this suggests the microbiota drives the effect of diet on intestinal inflammation, and that DFs may promote or mitigate inflammation in a microbiota-dependent manner.

While both Fer groups displayed reduced inflammation, we questioned why the feralized mice given the FR diet were slightly more protected against colitis than those deprived of DFs in terms of clinical outcomes and systemic inflammation markers, and why the two groups on a fiber-rich diet resulted in such diverging outcomes. Assessing the repertoire of predicted genes encoding DF- and mucus-degrading enzymes (CAZymes) in the MAGs revealed a higher abundance of DF-CAZymes in mice fed a fiber-rich diet, aligning with the differences observed between the different diet groups, but could not differentiate between Lab_FR_ and Fer_FR_ in most cases. However, assessing which MAGs that contributed with the specific CAZymes in the different groups revealed that the DF-degrading properties could be attributed to different species in the two groups. While the respective top contributors in Lab_FR_ and Fer_FR_—*L. muris* and *M. gordoncarteri*—are both relatively recently isolated species and little is known about their functions *in vivo*, ^85^^,^^86^ their implication in disease cannot be ruled out.

Moreover, the exact mechanism of colitis induction by DSS remains unresolved. While high doses of DSS may affect epithelial cells directly,^87^^,^^88^ the current understanding is that colitis occurs via microbial encroachment caused by weakening of the mucus layer by DSS.^89^ Bacterial penetration of the mucus is also observed in human patients with UC, further supporting the relevance of the use of DSS as a preclinical mouse model of colitis.^89^ The gut microbiota does not seem to be affected by DSS *per se*, ^90^ but are central to colitis development, as germ-free animals develop only mild and abnormal colitis.^87^^,^^88^ Chassaing et al. previously addressed how different floras affect DSS-induced colitis susceptibility,^87^ highlighting *Helicobacter*, a genus whose relevance to the gut barrier we have also emphasized previously.^16^

Indeed, the different outcomes following DSS-colitis presented herein may be explained by the presence or absence of specific taxa previously implicated in modulating DSS-colitis severity. R-Lab mice were enriched in *Duncaniella*, a genus encompassing species like *Duncaniella muricolitica* and *Duncaniella muris* that have been shown to induce severe weight loss, increase mortality, and enhance susceptibility to DSS-colitis.^91^^,^^92^ R-Fer mice showed reduced abundance of this genus, and were instead enriched in *Bacteroides* that contains several taxa associated with colitis protection, such as *Bacteroides uniformis*, *Bacteroides thetaiotaomicron*, and *Bacteroides acidifaciens.*^93‐95^ While *Duncaniella* at genus level was not differentially abundant in experiment 1, *D. muricolitica* was among the most abundant MAGs in Lab_FR._ Moreover, *Alistipes* was associated with Lab mice in both experiments and appeared among the most abundant MAGs in Lab groups, a genus in which colitis-aggravating species are also found.^91^ The genus *Phocaeicola* was associated with Fer mice in both experiments and likely represents the common mouse gut commensal *Phocaeicola vulgatus* (previously *Bacteroides vulgatus*). *P. vulgatus* is known as a notable degrader of complex carbohydrates,^96^ which was also evident from the MAG assigned to this species within most abundant MAGs in Fer_FL_. While *P. vulgatus* has also been implicated in alleviating colitis,^97^^,^^98^ its effects may be strain-specific.^99^ FL mice were particularly enriched in *Taurinivorans*, which only has one known species: *Taurinivorans muris*, a taurine-respiring bacterium capable of producing H_2_S using the DsrAB-DsrC system.^100^ *T. muris* therefore likely contributed to the increased genomic potential for *dsrAB* observed in FL. This could also align with the observed increase in bile acid deconjugating capacity (*bsh*), as the release of taurine from bile acids would support the growth of *T. muris*. H_2_S plays a dual role in gut health, as certain H_2_S levels can reduce the abundance of pathogenic taxa, but too high levels may promote inflammation and disturb the epithelial barrier.

Our findings of differentially abundant taxa dependent on intake of DFs argue that DFs applied a selection pressure on the microbiota, potentially driving divergent community structures in Lab_FR_ and Fer_FR_, ultimately influencing disease susceptibility through the presence, absence, or distinct distribution of specific bacterial species. Although additional analyzes, including metabolomics and proteomics, would be required to identify precise microbe–microbe and host‒microbe mechanisms, the current study demonstrates that DFs promote the establishment of a feralized microbiota. This resulting microbial profile appears to enhance gut barrier function in a manner that may support intestinal homeostasis. Furthermore, the results provide associations between specific taxa enriched in a naturalized microbiota and health-promoting effects, warranting targeted investigation in future work.

While this study demonstrates reproducible effects of feralization, it is important to consider the inherent limitations of the model. As the nature of the feralization model revolves around introducing an environment typically experienced by wild mice, a number of uncontrolled variables follow. Factors that may influence feralization include seasonal fluctuations and differences in the type of farm animals included, their diet, and microbial survival in regard to outdoor temperature and aerobic conditions. Moreover, the soil microbiome has the potential to affect gut microbiota and immunity in mice,^101^ but effects of nonmicrobial soil components are less studied. A number of inorganic soil constituents and certain bioactive compounds (e.g., antimicrobials) may both directly and indirectly engage with gut microbes and alter immune responses.^102^ Soil organic matter, such as humic and fulvic acids, can also modulate microbial communities.^103‐105^ Similar compounds, along with residual DFs, occur in farm animal feces, adding further variability. Collectively, this suggests that such non‑microbial elements may interact with and shape gut microbiome structure and gastrointestinal health. Nonetheless, this inherent variability is central to naturalization approaches, and findings across feralization studies remain consistent, as highlighted in a recent perspective.^18^ In line with this, the donors for the FMT were not the same mice as in experiment 1, recipients were not pre-treated with antibiotics or PEG, nor was food, water, or bedding sterilized, which is common practice in FMT experiments.^106^ Still, two separate experiments reproduced similar outcomes, arguing it was sufficient to introduce crucial taxa. Finally, findings in FMT recipients in this study and in comparable studies^7^^,^^107^ suggest that microbial exposure is the primary factor in these models.

FMT is a frequently used method to explore or demonstrate causality between the gut microbiota and its effects on the host. Recipient animals are commonly germ-free or administered antibiotics or laxatives prior to FMT to facilitate engraftment of the inoculum. In the present study, FMT was conducted in 3-week-old mice (at weaning) without any pretreatment. This approach was rooted in a number of considerations.^106^ First, GF mice lack the immunological cues from the microbiota that take place in early life.^108^ Second, antibiotics introduce confounding factors that may impede inoculum colonization, and laxatives potentially disrupt the gut environment by introducing oxygen and disturbing the mucus.^89^^,^^106^ Third, while our approach may reduce engraftment efficiency due to competition from the resident microbiota, the microbiota during the weaning period shows increased plasticity before a stable adult community is established,^109^ and FMT during early life is also shown to be more effective than in adulthood.^110^^,^^111^ Thus, performing FMT at weaning provides a biologically relevant compromise that avoids the confounding effects of depletion strategies while still allowing donor-associated microbial and phenotypic features to emerge.

In conclusion, we demonstrate that a fiber-rich diet alleviated colitis in feralized mice but exacerbated disease in clean laboratory mice. Although both groups showed similar potential to utilize DFs, FMT from feralized mice on a fiber-rich diet reproduced protection against DSS, highlighting the role of microbiota in modulating disease. These findings suggest that the beneficial effects of DFs are shaped by the broader ecological context of the host microbiome. To optimize the utilization of fiber-rich diets for improving human health, future research should pay close attention to which specific fibers are included in relation to microbial community dynamics. A strategic combination of distinct dietary fibers, gut microbiome profiles, and health states may reveal mechanisms by which the microbiome can be effectively modulated to enhance fiber utilization. This insight could pave the way for personalized dietary interventions that restore the optimal balance of the microbiota and improve outcomes in clinical settings.

## Supplementary Material

Supplementary Table S8.xlsx Supplementary Table S8.xlsx

Supplementary material.docx Supplementary material.docx

Supplementary Table S6.xlsx Supplementary Table S6.xlsx

Supplementary Table S5.xlsx Supplementary Table S5.xlsx

## Data Availability

The data underlying this article are available either in the Sequence Read Archive at https://www.ncbi.nlm.nih.gov/sra, and can be accessed with accession number PRJNA13205140, in its online supplementary material, or may be shared on reasonable request to the corresponding author.

## Abbreviations

LCN2: lipocalin-2
DF: dietary fiber
DSS: dextran sulfate sodium
B6: C57BL/6
Fer: feralized (mouse)
Lab: laboratory (mouse)
FL: fiber low
FR: fiber rich
FMT: fecal microbiota transfer
DAI: disease activity index
ASV: amplicon sequence variant
MAG: metagenome-assembled genome
CAZyme: carbohydrate active enzyme
MLG: mixed-linkage glucan
GH: glycoside hydrolase
CE: carbohydrate esterase
