# Supplementary material for: A naturalized gut microbiome interacts with dietary fibers to protect against colonic inflammation
Source: Gut Microbes. 2026 Mar 28;18(1):2649435. doi: 10.1080/19490976.2026.2649435 (PMC13034635; doi:10.1080/19490976.2026.2649435)
Supplement: Supplementary material.docx [file KGMI_A_2649435_SM1565.docx]

SUPPLEMENTARY MATERIAL

**Supplementary Table S1: Composition and proximates of special diets**. Provided by Ssniff Spezialdiäten GmbH. FL, fiber low; FR, fiber rich.

| **Ingredients** | **U** | **FL (AIN-93M)** | **FR** |
| --- | --- | --- | --- |
| Casein | % | 14.0000 | 11.8000 |
| L-Cysteine | % | 0.1800 | 0.1800 |
| Corn starch | % | 46.5892 | 39.2692 |
| Maltodextrin | % | 15.5000 | 15.5000 |
| Sucrose | % | 10.0000 | 10.0000 |
| Cellulose | % | 5.0000 | -- |
| Fiber mix | % | -- | 15.1200 |
| Vitamin premixture | % | 1.0000 | 1.0000 |
| Mineral mix-1 | % | 3.5000 | -- |
| Mineral mix-2, mix adjusted | % | -- | 3.5000 |
| Choline bitartrate | % | 0.2300 | 0.2300 |
| t-BHQ | % | 0.0008 | 0.0008 |
| Soybean oil | % | 4.0000 | 3.4000 |
| **Proximate contents** |  |  |  |
| Crude protein | % | 12.3 | 12.6 |
| Crude fat | % | 4.1 | 4.1 |
| Total dietary fiber | % | -- | 5.0 |
| Crude ash | % | 3.1 | 3.7 |
| Starch | % | 44.8 | 42.5 |
| Sugar | % | 11.2 | 11.2 |
| **Amino acids** |  |  |  |
| Lysine | % | 1.03 | 0.95 |
| Methionine | % | 0.38 | 0.36 |
| Met+Cys | % | 0.61 | 0.62 |
| Threonine | % | 0.54 | 0.53 |
| Tryptophan | % | 0.16 | 0.17 |
| **Minerals** |  |  |  |
| Calcium | % | 0.55 | 0.57 |
| Phosphorus | % | 0.36 | 0.45 |
| Sodium | % | 0.16 | 0.16 |
| Magnesium | % | 0.09 | 0.17 |
| Potassium | % | 0.54 | 0.59 |
| ME (Atwater) * | MJ/kg | 15.6 | 15.6 |
| Protein | kcal% | 13 | 14 |
| Fat | kcal% | 10 | 10 |
| Carbohydrates | kcal% | 77 | 76 |

* Physiological fuel value

**Supplementary Table S2A**: Disease Activity Index (DAI) score sheets for experiment 1.

| **Feature** | **Score** | **Description** |
| --- | --- | --- |
| Body weight loss | 0 | <5% |
|  | 1 | 5-15% |
|  | 5 | 15-20% |
|  | 10 | >20% |
| Feces quality | 0 | Normal, no blood |
|  | 1 | Loose and/or blood traces |
|  | 3 | Fluid and/or blood |
| Activity level | 0 | Normal |
|  | 1 | Full mobility, less active |
|  | 3 | Little/no movement, little/no reaction to touch |
| Body posture | 0 | Normal |
|  | 1 | Slight hunching |
|  | 3 | Clear hunching |

**Supplementary Table S2B**: Disease Activity Index (DAI) score sheets for experiment 2.

| **Feature** | **Score** | **Description** |
| --- | --- | --- |
| Body weight loss | 0 | <5% |
|  | 2 | 5-10% |
|  | 5 | 11-15% |
|  | 7 | 16-20% |
|  | 10 | >20% |
| Feces quality | 0 | Normal |
|  | 1 | Somewhat loose and/or small blood traces |
|  | 5 | Loose and blood traces |
|  | 10 | Fluid and/or blood |
| Activity level | 0 | Normal |
|  | 2 | Slightly reduced |
|  | 5 | Considerably reduced |
|  | 10 | Severe inactivity, little/no reaction to touch |
| Appearance | 0 | Normal |
|  | 2 | Slight piloerection |
|  |  |  |
|  | 7 | Hunchback and piloerection |
| Grimace scale^†^ | 0 | Not present |
|  | 5 | Moderate |
|  | 10 | Obvious |

† <https://www.nc3rs.org.uk/3rs-resources/grimace-scales/grimace-scale-mouse>

**Supplementary Table S3: Absorbance ratios of RNA** samples in experiment 1. A230, absorbance at 230 nm; A260, absorbance at 260 nm; A280, absorbance at 280 nm.

| **Mucosa** | | | | |  | **Liver** | | | | |
| --- | --- | --- | --- | --- | --- | --- | --- | --- | --- | --- |
| **Sample#** | **A260/280; A260/230** |  | **Sample#** | **A260/280; A260/230** |  | **Sample#** | **A260/280; A260/230** |  | **Sample#** | **A260/280; A260/230** |
| 1 | 2.11; 2.24 |  | 30 | 2.11; 2.18 |  | 1 | 2.13; 2.07 |  | 29 | 2.14; 2.21 |
| 2 | 2.11; 2.05 |  | 31 | 2.11; 2.17 |  | 2 | 2.14; 2.18 |  | 30 | 2.12; 2.1 |
| 3 | 2.09; 2.22 |  | 32 | 2.11; 2.24 |  | 3 | 2.1; 1.9 |  | 31 | 2.13; 2.17 |
| 4 | 2.12; 2.2 |  | 33 | 2.12; 2.19 |  | 4 | 2.12; 2.16 |  | 32 | 2.12; 2.09 |
| 5 | 2.11; 2.23 |  | 34 | 2.11; 2.19 |  | 5 | 2.11; 2.01 |  | 33 | 2.12; 2.04 |
| 6 | 2.1; 2.23 |  | 35 | 2.11; 2.18 |  | 6 | 2.13; 2.05 |  | 34 | 2.13; 2.16 |
| 7 | 2.11; 2.19 |  | 36 | 2.09; 2.2 |  | 7 | 2.13; 2.02 |  | 35 | 2.12; 2.14 |
| 8 | 2.1; 2.19 |  | 37 | 2.13; 2.18 |  | 8 | 2.13; 2.21 |  | 36 | 2.14; 2.2 |
| 9 | 2.13; 2.19 |  | 38 | 2.11; 2.22 |  | 9 | 2.13; 2.19 |  | 37 | 2.12; 2.11 |
| 10 | 2.11; 2.06 |  | 39 | 2.13; 2.21 |  | 10 | 2.12; 2.13 |  | 38 | 2.12; 2.08 |
| 11 | 2.15; 1.15 |  | 40 | 2.12; 2.23 |  | 11 | 2.11; 2.06 |  | 39 | 2.09; 2.09 |
| 12 | 2.11; 2.22 |  | 41 | 2.12; 2.16 |  | 12 | 2.13; 2.15 |  | 40 | 2.14; 2.2 |
| 13 | 2.11; 2.23 |  | 42 | 2.09; 2.14 |  | 13 | 2.14; 2.18 |  | 41 | 2.05; 1.88 |
| 14 | 2.1; 2.17 |  | 43 | 2.12; 2.22 |  | 14 | 2.12; 2.15 |  | 42 | 2.11; 2.03 |
| 15 | 2.11; 2.16 |  | 44 | 2.13; 2.22 |  | 15 | 2.14; 2.19 |  | 43 | 2.14; 2.19 |
| 16 | 2.12; 2.09 |  | 45 | 2.12; 2.22 |  | 16 | 2.13; 2.18 |  | 44 | 2.13; 2.2 |
| 17 | 2.13; 2.21 |  | 46 | 2.1; 2.23 |  | 17 | 2.1; 2.06 |  | 45 | 2.11; 2.11 |
| 18 | 2.09; 2.2 |  | 47 | 2.13; 2.14 |  | 18 | 2.12; 2.12 |  | 46 | 2.11; 2.08 |
| 20 | 2.11; 2.17 |  | 48 | 2.12; 2.25 |  | 19 | 2.11; 2.06 |  | 47 | 2.13; 2.16 |
| 21 | 2.14; 2.24 |  | 49 | 2.11; 2.05 |  | 20 | 2.1; 1.92 |  | 48 | 2.12; 2.16 |
| 22 | 2.1; 2.22 |  | 50 | 2.12; 2.23 |  | 21 | 2.11; 2.12 |  | 49 | 2.13; 2.18 |
| 23 | 2.11; 2.18 |  | 51 | 2.13; 2.25 |  | 22 | 2.12; 2.14 |  | 50 | 2.14; 2.19 |
| 24 | 2.12; 2.18 |  | 52 | 2.12; 2.21 |  | 23 | 2.13; 2.17 |  | 51 | 2.14; 2.1 |
| 25 | 2.12; 2.22 |  | 53 | 2.12; 2.13 |  | 24 | 2.14; 2.2 |  | 52 | 2.11; 2.02 |
| 26 | 2.12; 2.22 |  | 54 | 2.13; 2.04 |  | 25 | 2.1; 1.96 |  | 53 | 2.14; 2.16 |
| 27 | 2.13; 2.13 |  | 55 | 2.11; 2.18 |  | 26 | 2.1; 1.94 |  | 54 | 2.13; 2.16 |
| 28 | 2.13; 2.2 |  |  |  |  | 27 | 2.15; 2.17 |  | 55 | 2.12; 2.15 |
| 29 | 2.12; 2.16 |  |  |  |  | 28 | 2.14; 2.2 |  |  |  |

**Supplementary Table S4**: **Primer sequences** of target genes used for RT-qPCR of RNA extracted from the liver and the colon mucosa.

| **Protein** | **Target gene** | **Tissue** | **Primer** | **5’ – 3’ sequence** |
| --- | --- | --- | --- | --- |
| TATA-binding protein | *Tbp* | Liver | Forward | CCTTGTACCCTTCACCAATGAC |
|  |  |  | Reverse | ACAGCCAAGATTCACGGTAGA |
| Glyceraldehyde-3-phosphate dehydrogenase | *Gapdh* | Liver | Forward | CTTCAACAGCAACTCCCACTCTT |
|  |  |  | Reverse | GCCGTATTCATTGTCATACCAGG |
| Interleukin-1β | *Il1b* | Liver | Forward | GCAGCTGGAGAGTGTGGAT |
|  |  |  | Reverse | AAACTCCACTTTGCTCTTGACTT |
| Alkaline phosphatase, intestinal | *Alpi* | Mucosa | Forward | TCGCCACTCAACTCATCTCC |
|  |  |  | Reverse | AGTCCCCTTGGGAAACATGAA |
| Angiogenin 4 | *Ang4* | Mucosa | Forward | CTCCAGGAGCACACAGCTA |
|  |  |  | Reverse | CAGCACGAAGACCAACAACA |
| Cadherin-1 | *Cdh1* | Mucosa | Forward | ATTGCAAGTTCCTGCCATCC |
|  |  |  | Reverse | CAGTAGGAGCAGCAGGATCA |
| Calcium-activated chloride channel regulator 1 | *Clca1* | Mucosa | Forward | ACAACCACTAAGGTGGCCTA |
|  |  |  | Reverse | GAGCTCGCTTGAATGCTGTA |
| Alpha-defensin 24 | *Defa24* | Mucosa | Forward | CAGAAGGCGCTTCTCTTCAA |
|  |  |  | Reverse | TTTGCAGCCTCTTGCTCTAC |
| Dual oxidase 2 | *Duox2* | Mucosa | Forward | GGCAGCCAGATGCTCTGTAA |
|  |  |  | Reverse | ATGTCAGCCAGCCACTCAAA |
| F11 receptor | *F11r* | Mucosa | Forward | TGGAGTGGAAGTTCGTCCAA |
|  |  |  | Reverse | AGGTGACTCGGTCCGCATA |
| Fc gamma binding protein | *Fcgbp* | Mucosa | Forward | ATCGAGCAATGTGGCTGCTA |
|  |  |  | Reverse | CAATGCTGCTGGCAGTTTTCA |
| Glyceraldehyde-3-phosphate dehydrogenase | *Gapdh* | Mucosa | Forward | CAAGGTCATCCCAGAGCTGAA |
|  |  |  | Reverse | CAGATCCACGACGGACACA |
| Gasdermin C | *Gsdmc* | Mucosa | Forward | AGGTTCAGAGTAAGAGCATCCC |
|  |  |  | Reverse | ATGTGGGCAACTGATCCAAC |
| Gasdermin D | *Gsdmd* | Mucosa | Forward | GAGCCCAGTGCTCCAGAA |
|  |  |  | Reverse | TGTTCCCATCGACGACATCA |
| Heme oxygenase 1 | *Hmox1* | Mucosa | Forward | TCAAGCACAGGGTGACAGAA |
|  |  |  | Reverse | ATCACCTGCAGCTCCTCAAA |
| Interleukin-10 | *Il10* | Mucosa | Forward | AAAGGACCAGCTGGACAACA |
|  |  |  | Reverse | TAAGGCTTGGCAACCCAAGTA |
| Interleukin-18 | *Il18* | Mucosa | Forward | CAAAGAAAGCCGCCTCAAAC |
|  |  |  | Reverse | GACGCAAGAGTCTTCTGACA |
| Interleukin-1b | *Il1b* | Mucosa | Forward | TGGCAACTGTTCCTGAACTCA |
|  |  |  | Reverse | GGGTCCGTCAACTTCAAAGAAC |
| Interleukin-25 | *Il25* | Mucosa | Forward | CTCTCTCAGAAGGCCTGTCA |
|  |  |  | Reverse | CCCACGATCATTGCCAAGAA |
| Intelectin-1 | *Itln1* | Mucosa | Forward | TCTTTTCCTCTCTGCCCAGAA |
|  |  |  | Reverse | GTGCGCAGGAAATAGAGACC |
| Lipocalin 2 | *Lcn2* | Mucosa | Forward | GCTACAATGTCACCTCCATCC |
|  |  |  | Reverse | CCCTGGAGCTTGGAACAAA |
| Mucin 2 | *Muc2* | Mucosa | Forward | CAGCACACCAACCAAAACCA |
|  |  |  | Reverse | CACAGCCACCAGGTCTCATTA |
| Mucin 3 | *Muc3* | Mucosa | Forward | CCGGAGTATGAAGGGGTTATCA |
|  |  |  | Reverse | ACTTGGCCTTCAGGATGACA |
| Myosin light chain kinase | *Mylk* | Mucosa | Forward | TTCAACAGGGTCACCAACCA |
|  |  |  | Reverse | TCCAGGAAAGCTTGGGAGAC |
| NOD-like receptor family pyrin domain containing 6 | *Nlrp6* | Mucosa | Forward | CACCTCGGTGCTTCTCTCC |
|  |  |  | Reverse | TTCACCTTAGCATGCTGTCGTA |
| Nucleotide-binding oligomerization domain 1 | *Nod1* | Mucosa | Forward | GTGGCTTTGGCTGTGAAGAA |
|  |  |  | Reverse | TTTGCCCCTTCGTCTCCAA |
| Nucleotide-binding oligomerization domain 2 | *Nod2* | Mucosa | Forward | AAGCCCTGGCTGAAGTTGTA |
|  |  |  | Reverse | CATGCTGCCAATGTTGTTTCC |
| Nitric oxide synthase 2 | *Nos2* | Mucosa | Forward | GAGGAGCAGGTGGAAGACTA |
|  |  |  | Reverse | GGAAAAGACTGCACCGAAGATA |
| NADPH oxidase 1 | *Nox1* | Mucosa | Forward | GTGCCGACAACAAGCTCAAA |
|  |  |  | Reverse | GCAAAGGCACCTGTCTCTCTA |
| NAD(P)H quinone dehydrogenase 1 | *Nqo1* | Mucosa | Forward | AAGCTGCAGACCTGGTGATA |
|  |  |  | Reverse | ACGAGCACTCTCTCAAACCA |
| Occludin | *Ocln* | Mucosa | Forward | GAATGGCAAGCGATCATACCC |
|  |  |  | Reverse | GAATCTCCTGGGCCACTTCA |
| Regenerating islet-derived 3 beta | *Reg3b* | Mucosa | Forward | CTTTCTGTGGCAGCTTGTCA |
|  |  |  | Reverse | TAGGGCAACTTCACCTCACA |
| Regenerating islet-derived 3 gamma | *Reg3g* | Mucosa | Forward | GTATGGATTGGGCTCCATGAC |
|  |  |  | Reverse | CATCAGCATTGCTCCACTCC |
| Resistin-like beta | *Retnlb* | Mucosa | Forward | CCTAAGACGATCTCCTGCACTA |
|  |  |  | Reverse | AGCACATCCAGTGACAACCA |
| Serum amyloid A1 | *Saa1* | Mucosa | Forward | ATCTCTCATGTGTGTATCCCACAA |
|  |  |  | Reverse | TACCCTCTCCTCCTCAAGCA |
| TATA-binding protein | *Tbp* | Mucosa | Forward | ACCAGAACAACAGCCTTCCA |
|  |  |  | Reverse | AAAGATGGGAATTCCAGGAGTCA |
| Transforming growth factor beta 1 | *Tgfb1* | Mucosa | Forward | GCTGCGCTTGCAGAGATTAA |
|  |  |  | Reverse | GTAACGCCAGGAATTGTTGCTA |
| Tight junction protein 1 | *Tjp1* | Mucosa | Forward | TCTGGCATCATTCGCCTTCA |
|  |  |  | Reverse | TCAACCGCATTTGGCGTTAC |
| Toll-like receptor 2 | *Tlr2* | Mucosa | Forward | TGCATCACCGGTCAGAAAAC |
|  |  |  | Reverse | AGCCAAAGAGCTCGTAGCA |
| Toll-like receptor 4 | *Tlr4* | Mucosa | Forward | GTTCTTCTCCTGCCTGACAC |
|  |  |  | Reverse | GCTGAGTTTCTGATCCATGCA |
| Toll-like receptor 5 | *Tlr5* | Mucosa | Forward | ATGGATGGATGCTGAGTTCCC |
|  |  |  | Reverse | CTGGCCATGAAGATCACACCTA |
| Z-DNA binding protein 1 | *Zbp1* | Mucosa | Forward | TGGCAGAAGCTCCTGTTGAC |
|  |  |  | Reverse | CCAGCTGGCCAATCTTCACA |

**Supplementary Table S5:** colonic mucosa gene expression analysis statistics (sheet 1) and raw data (sheet 2) (.xlsx file).

**Supplementary Table S6:** Predicted pathways and genes encoding CAZymes involved in xylan, β-glucan, and mucin degradation in the fecal microbiome, staistics (sheet 1+3) and sample-wise mean abundance (sheet 2+4) (.xlsx file).

**Supplementary Table S7**: Top contributing species to each CAZy family. Fer, feralized; Lab, laboratory; FR, fiber rich; FL, fiber low; GH, glycoside hydrolase; CE, carbohydrate esterase.

| **CAZyme family** | **Top contributing species** | | | |
| --- | --- | --- | --- | --- |
|  | **Fer_FL_** | **Fer_FR_** | **Lab_FL_** | **Lab_FR_** |
| CE2 | *s_Alistipes sp002358415* | *s_Alistipes sp002358415* | *s_Alistipes sp002358415* | *s_Alistipes sp002358415* |
| CE4 | *g_Gallimonas* | *s_CAG-269 sp948441945* | *s_Kineothrix sp910588855* | *s_COE1 sp009774245* |
| CE6 | *s_Anaerotignum sp910576545* | *s_Lepagella muris* | *s_Fimiplasma sp910588165* | *s_Lepagella muris* |
| CE7 | *s_Bacteroides acidifaciens* | *s_Muribaculum gordoncarteri* | *s_Alistipes sp002362235* | *s_Bacteroides acidifaciens* |
| GH1 | *s_Angelakisella sp910585535* | *s_Faecalibaculum rodentium* | *s_Kineothrix sp910588855* | *s_Faecalibaculum rodentium* |
| GH10 | *s_Akkermansia muciniphila* | *s_Muribaculum gordoncarteri* | *s_Acetatifactor sp011959105* | *s_Lepagella muris* |
| GH101 | *s_CAJFPI01 sp022801715* | *s_MGBC124762 sp910579775* | *s_CAJFPI01 sp022801715* | *s_Roseburia_B sp910585825* |
| GH11 | *s_COE1 sp002358575* | *s_Butyribacter sp009774235* | *s_COE1 sp002358575* | *s_COE1 sp002358575* |
| GH115 | *s_Akkermansia muciniphila* | *s_Muribaculum gordoncarteri* | *s_Acetatifactor sp011959105* | *s_Lepagella muris* |
| GH129 | *s_Acetatifactor sp948592185* | *s_Pullibacteroides sp947176875* | *s_Acetatifactor sp910584865* | *s_Pullibacteroides sp947176875* |
| GH16 | *s_Alistipes sp002358415* | *s_Alistipes sp002358415* | *s_Cryptobacteroides sp910585445* | *s_Alistipes sp002358415* |
| GH2 | *s_Alistipes sp002358415* | *s_JAGBWK01 sp947174275* | *s_Cryptobacteroides sp910585445* | *s_Lepagella muris* |
| GH20 | *s_Alistipes sp002358415* | *s_UBA3263 sp001689615* | *s_Cryptobacteroides sp910585445* | *s_Alistipes sp002358415* |
| GH29 | *s_Alistipes sp002358415* | *s_Paramuribaculum intestinale* | *s_Cryptobacteroides sp910585445* | *s_Alistipes sp002358415* |
| GH3 | *s_Akkermansia muciniphila* | *s_JAGBWK01 sp947174275* | *s_Cryptobacteroides sp910585445* | *s_Cryptobacteroides sp910585445* |
| GH33 | *s_Alistipes sp002358415* | *s_UBA3263 sp001689615* | *s_Cryptobacteroides sp910585445* | *s_Alistipes sp002358415* |
| GH35 | *s_Akkermansia muciniphila* | *s_Bacteroides acidifaciens* | *s_Eubacterium_R sp011958665* | *s_Bacteroides acidifaciens* |
| GH4 | *s_Acetatifactor sp011959105* | *s_Acetatifactor sp011959105* | *s_Acetatifactor sp011959105* | *s_Caccenecus sp910585045* |
| GH42 | *s_Akkermansia muciniphila* | *s_UBA3282 sp009774585* | *s_Angelakisella sp910585535* | *s_COE1 sp009774245* |
| GH43 | *s_Akkermansia muciniphila* | *s_Muribaculum gordoncarteri* | *s_Acetatifactor sp011959105* | *s_Lepagella muris* |
| GH48 | *s_Aphodocola sp910580135* | *s_UBA3282 sp910577735* | *s_UBA3282 sp910577735* | *s_UBA3282 sp910577735* |
| GH5 | *s_Akkermansia muciniphila* | *s_Bacteroides muris* | *s_Cryptobacteroides sp910585445* | *s_Lepagella muris* |
| GH67 | *s_Akkermansia muciniphila* | *s_Muribaculum gordoncarteri* | *s_Akkermansia muciniphila* | *s_Lepagella muris* |
| GH84 | *s_Alistipes sp002358415* | *s_UBA3263 sp001689615* | *s_Alistipes sp002358415* | *s_UBA3263 sp001689615* |
| GH85 | *s_UBA3263 sp001689615* | *s_UBA3263 sp001689615* | *s_Lactobacillus taiwanensis* | *s_UBA3263 sp001689615* |
| GH89 | *s_Alistipes sp002358415* | *s_UBA3263 sp001689615* | *s_Cryptobacteroides sp910585445* | *s_Cryptobacteroides sp910585445* |
| GH95 | *s_Akkermansia muciniphila* | *s_JAGBWK01 sp947174275* | *s_Cryptobacteroides sp910585445* | *s_Bacteroides acidifaciens* |
| GH98 | *s_Desulfovibrio sp009773975* | *s_Corynebacterium stationis* | *s_MGBC162267 sp948552115* | *s_Corynebacterium stationis* |

**Supplementary Table S8**: non-CAZyme predicted genes statistics (sheet 1) and sample-wise mean relative abundance (sheet 2) (.xlsx file).
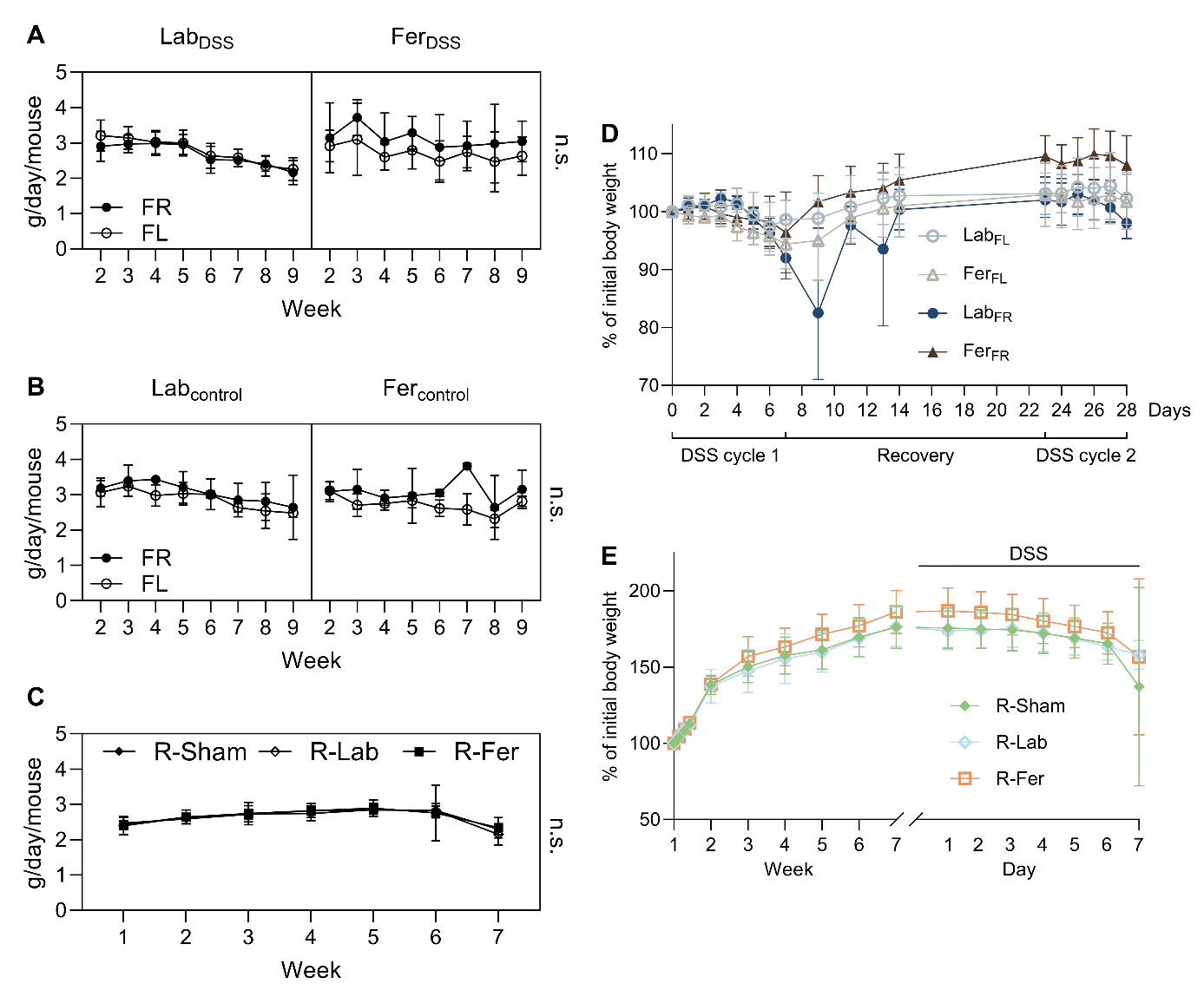


**Supplementary Figure S1: Average daily food intake per mouse in grams**. **A + B**) experiment 1 and **C**) experiment 2**.** Weighed weekly cage-wise **A**) DSS-treated n=2-4 cages, and **B**) controls n=2 cages; **C**) experiment 2: n=5 cages, all with 2-3 mice/cage. Difference in intake between the two diets were determined by three-way repeated measures ANOVA for DSS-treated animals and control groups separately, including only cages for which intake was recorded every week throughout the experiment. For experiment two, differences were determined by two-way repeated measures ANOVA. In all tests, Geisser-Greenhouse correction was applied. **D**) Body weight relative to initial weight during DSS-treated mice in experiment 1. **E**) Weekly body weight relative to initial for FMT recipient mice in experiment 2 including daily measurements during DSS-treatment in week 7. FL, fiber low; FR, fiber rich; Lab, laboratory; Fer, feralized; R, recipient; DSS, dextran sulfate sodium; n.s., not significant. Error bars, standard deviation.


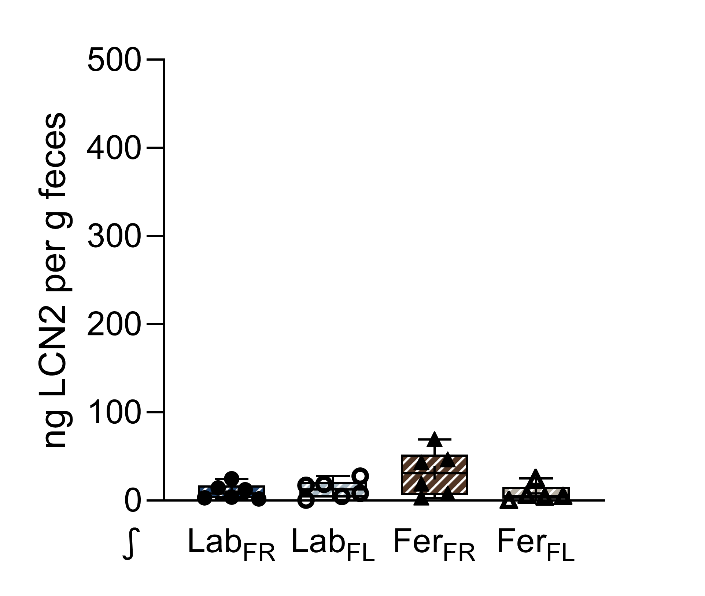


**Supplementary Figure S2: Fecal LCN2 in untreated animals**. No significant difference between groups (α = 0.05) determined by two-way ANOVA. Box plots show individual mice (dots/triangles), median (line), mean (+), IQR (box) and minimum to maximum (whiskers). Dark blue/filled dots, Lab_FR_ (n=6); light blue/open dots, Lab_FL_ (n=6); brown/filled triangles, Fer_FR_ (n=6); beige/open triangles, Fer_FL_ (n=5). Lab, laboratory; Fer, feralized; FR, fiber rich; FL, fiber low. Statistics performed on transformed data: ∫, log10.


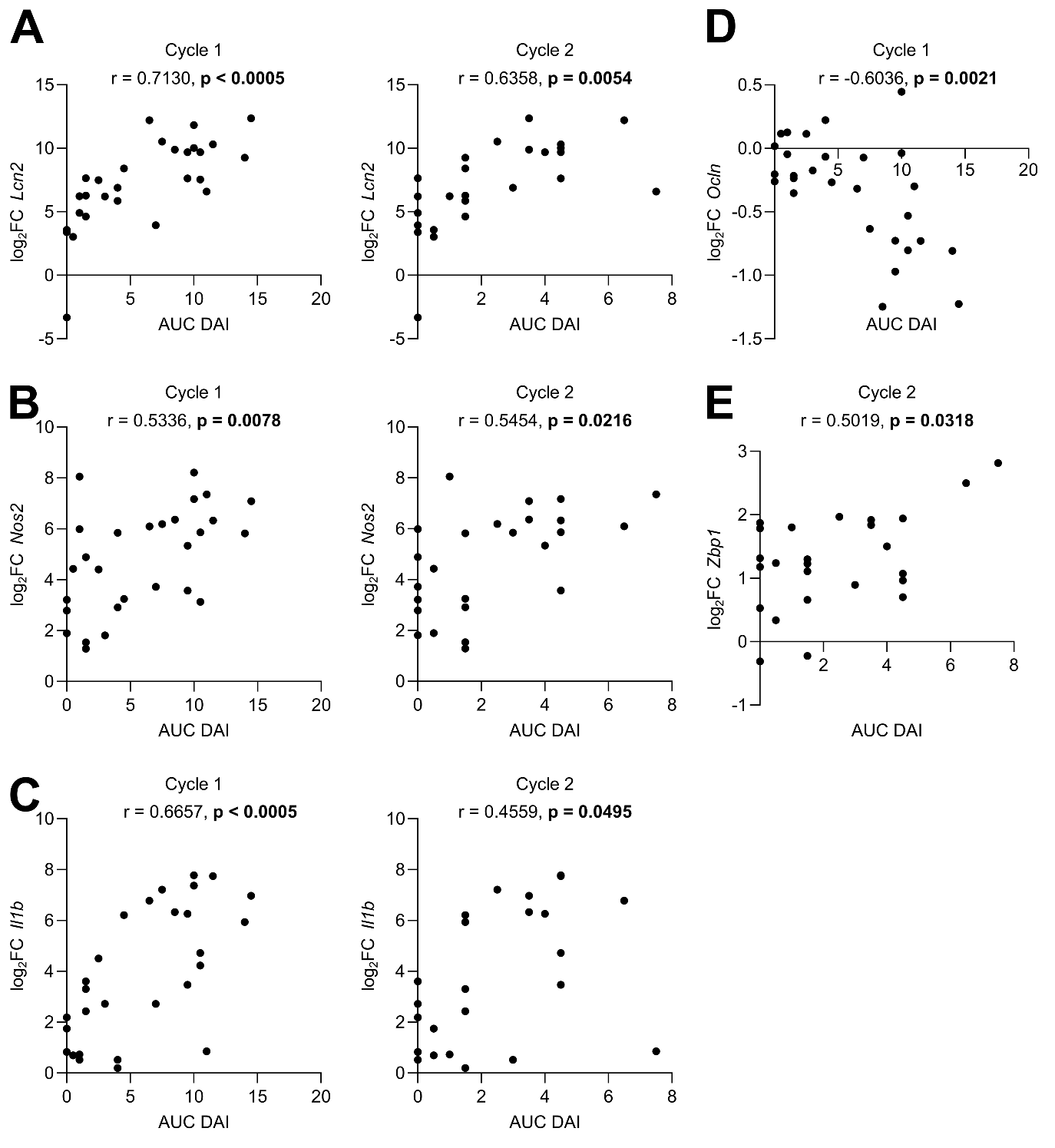


**Supplementary Figure S3: Correlation analysis of disease scoring and colonic gene expression**. Pearson correlation coefficient (r) calculated between area under the curve (AUC) of disease activity index (DAI) and relative expression of **A**) *Lcn2*, **B**) *Nos2*, and **C**) *Il-1b* for cycle 1 (left) and cycle 2 (right) of DSS-treatment. Pearson correlation coefficient (r) calculated between **D**) AUC of DAI in cycle 1 and relative expression of Ocln, **E**) AUC of DAI in cycle 2 and relative expression of *Zbp1*. The p-values were adjusted using Benjamini-Hochberg correction.


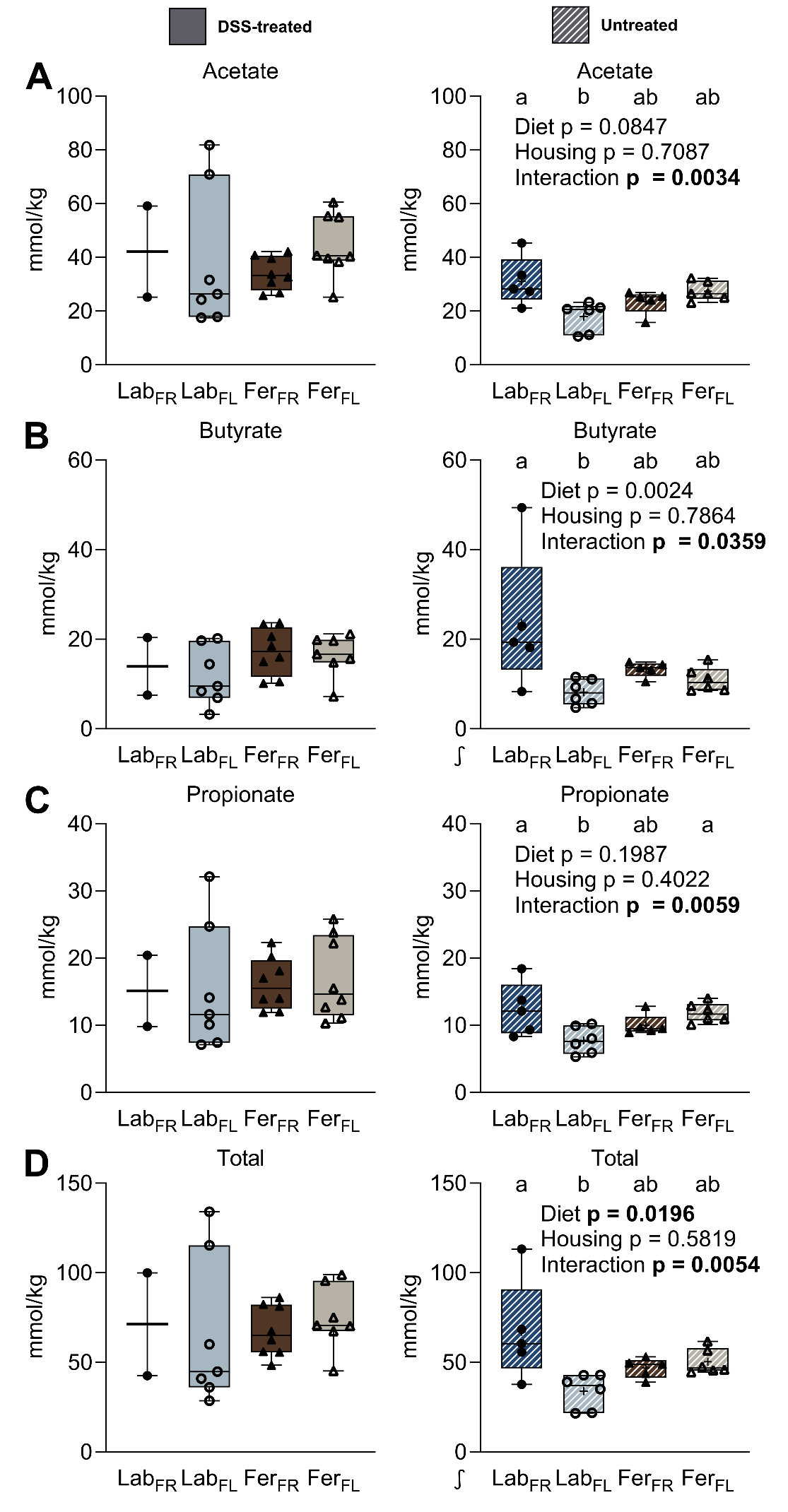


**Supplementary Figure S4: Cecal levels of short-chain fatty acids at termination,** **experiment 1**. Levels of **A**) acetate, **B**) butyrate, **C**) propionate, and **D**) total given in mmol/kg cecal contents. Levels in DSS-treated groups and untreated animals are given in the left and right panels, respectively. Dark blue/filled dots, Lab_FR_ (n=2); light blue/open dots, Lab_FL_ (n=7); brown/filled triangles, Fer_FR_ (n=8); beige/open triangles, Fer_FL_ (n=7-8). Untreated groups are n=5 or n=6 for FR and FL, respectively. Box plots show median (line), mean (+), IQR (box) and minimum to maximum (whiskers). p-values for the effect of diet, housing condition, and interaction determined by two-way ANOVA are given, and letters designate significant differences between groups following Tukey multiple comparison testing (adjusted p-value ≤ 0.05). Lab, laboratory; Fer, feralized; FR. fiber rich; FL, fiber low. ∫, Statistics performed on log_10_-transformed data.

**
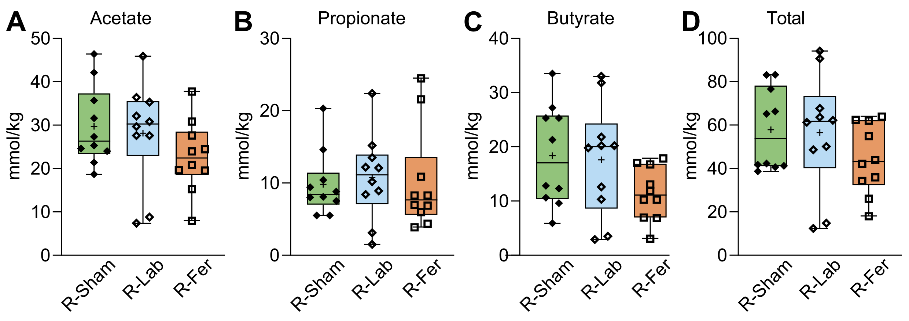
**

**Supplementary Figure S5: Cecal levels of short-chain fatty acids** **after DSS-treatment, experiment 2**. **A**) acetate, **B**) propionate, **C**) butyrate, and **D**) total levels given in mmol/kg. Green/filled diamonds, R-Sham; blue/open diamonds, R-Lab; orange/open square, R-Fer. n=10 for all groups. Box plots show median (line), mean (+), IQR (box) and minimum to maximum (whiskers). Significance of group means assessed by one-way ANOVA (all were non-significant, i.e., p>0.05). R, recipient; Lab, laboratory; Fer, feralized.

**
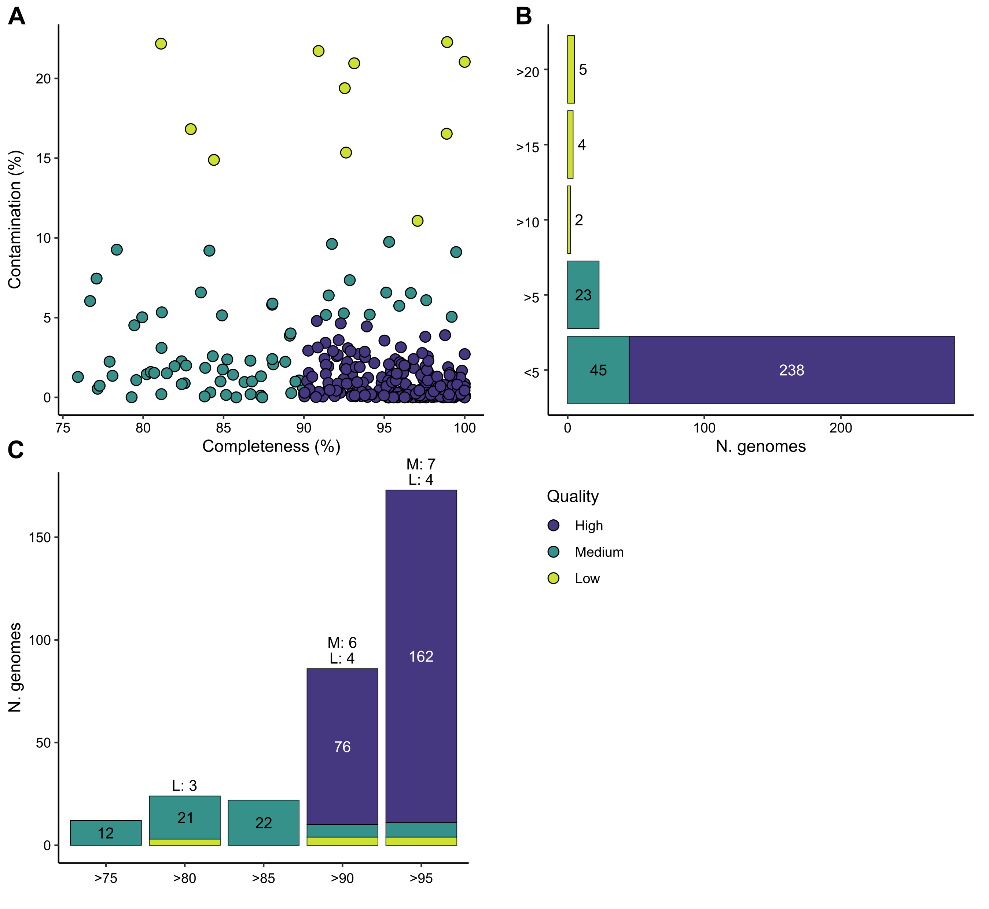
**

**Supplementary Figure S6: Metagenome-assembled genome (MAG) quality scores**. Distribution of quality scores estimated using checkM2. Colors and corresponding quality category according to MIMAG [^94^](#_ENREF_94): purple, high (>90% completeness and <5% contamination); teal, medium (≤50% completeness and <10% contamination); lime green, low-quality (<50% completeness and <10% contamination*). *11 low-quality MAGs in this dataset had 10<25% contamination. **A**) The dots represent MAGs that were deemed of sufficient quality and included in analyses. **B**) The horizontal stacked bar plot shows the distribution of the three categories of MAG quality (low, medium, and high) within the range of contamination scores. **C**) The vertical stacked bar plot illustrates the distribution of the three categories of MAG quality in the range of completeness scores. The number of genomes (N. genomes) is indicated by values inside, adjacent to, or above the boxes in **B**+**C**. L, low quality; M: medium quality.


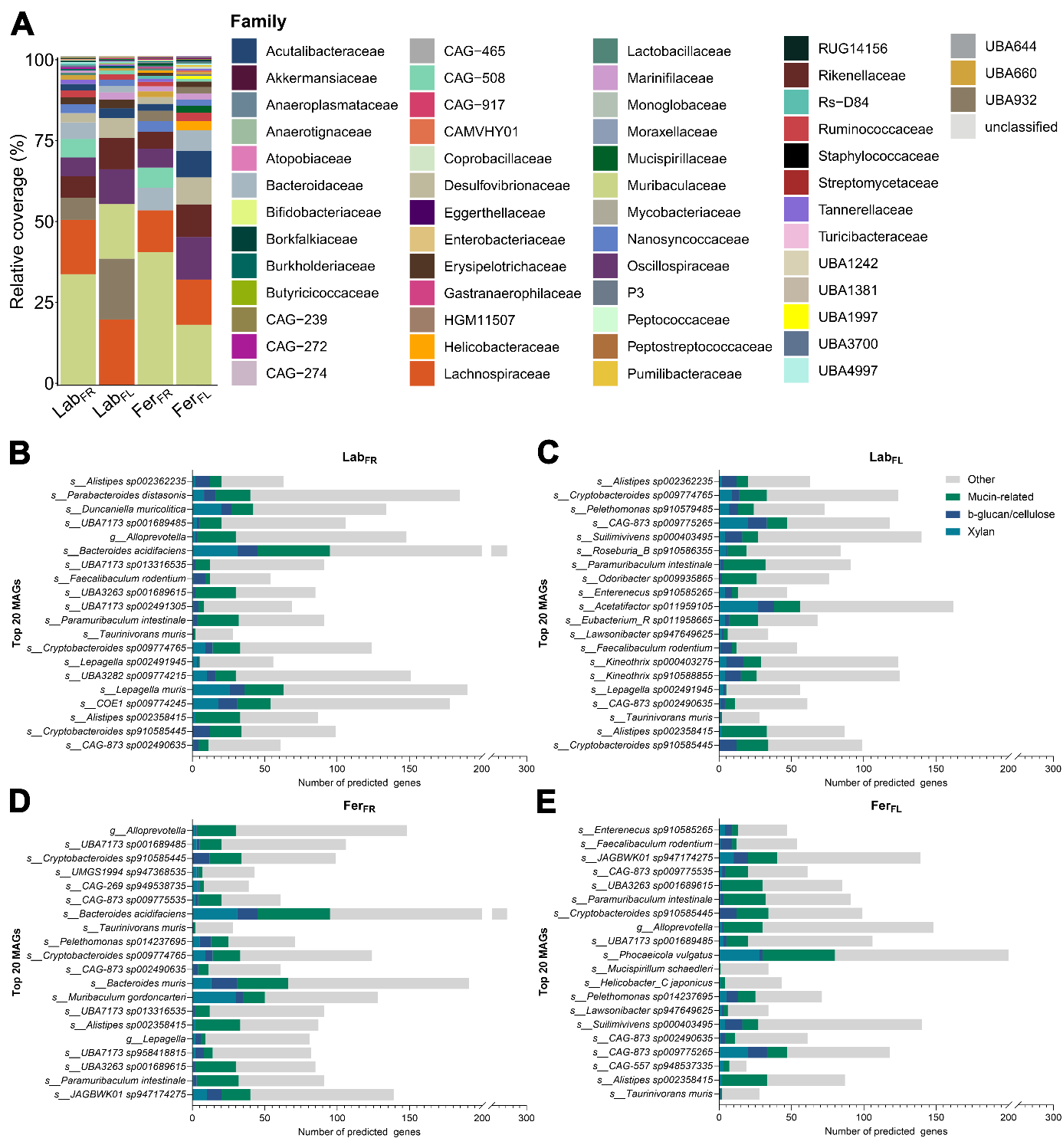
**Supplementary Figure S7: Abundance of bacterial MAGs in the fecal microbiota and their potential to degrade complex carbohydrates. A**) Group mean (n=5/group) relative abundance of MAGs binned at family level. **B**-**E**) Twenty highest relative abundance MAGs and the distribution of CAZyme genes predicted in these genomes within each group, **B**) LabFR, **C**) LabFL, **D**) FerFR, and **E**) FerFL (n=5/group). MAG, metagenome assembled genome; CAZyme, carbohydrate-active enzyme; Lab, laboratory; Fer, feralized; FL, fiber low; FR, fiber rich.


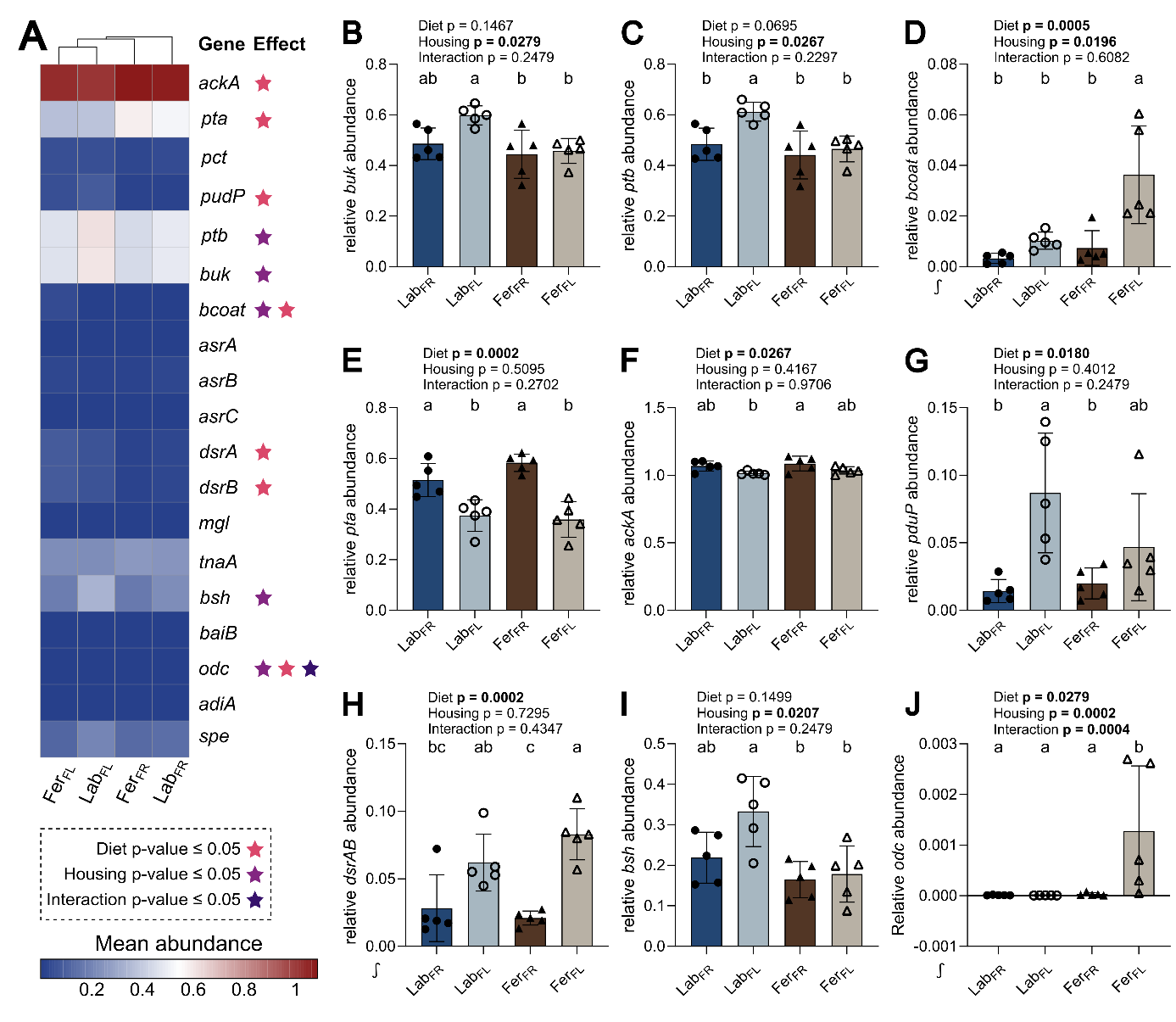


**Supplementary Figure S8: Mean relative abundance of predicted genes encoding enzymes involved in microbial metabolite production**. **A**) Heatmap indicating mean relative gene abundance (dot product of relative abundance and gene counts within MAGs) per group. plotted by hierarchical clustering using average linkage of Canberra distances. **B-J**) Bar plots of genes that were significantly affected by diet, housing condition, or their interaction, determined by two-way ANOVA (Benjamini-Hochberg adjusted p-values ≤ 0.05), and letters designate significant differences between groups following Tukey multiple comparison testing (adjusted p-value ≤ 0.05). Bar plots show individual mice (dots/triangles), mean (bar) and SD (whiskers). Dark blue/filled dots, Lab_FR_; light blue/open dots, Lab_FL_; brown/filled triangles, Fer_FR_; beige/open triangles, Fer_FL_. n=5 for all groups. ∫, statistics performed on log_10_- transformed data. Lab, laboratory; Fer, feralized; FR, fiber rich; FL, fiber low. See **Supplementary Table S8** for additional information on genes, statistics, and abundance values.
